# Sub-lethal oxidative stress induces lysosome biogenesis via a lysosomal membrane permeabilization-cathepsin-caspase 3-transcription factor EB-dependent pathway

**DOI:** 10.18632/oncotarget.14016

**Published:** 2016-12-18

**Authors:** San Min Leow, Shu Xian Serene Chua, Gireedhar Venkatachalam, Liang Shen, Le Luo, Marie-Veronique Clement

**Affiliations:** ^1^ Department of Biochemistry, Yong Loo Lin School of Medicine, National University of Singapore, Singapore, Singapore; ^2^ National University of Singapore Graduate School for Integrative Sciences and Engineering, Singapore, Singapore; ^3^ Biostatistic Unit, Yong Loo Lin School of Medicine, National University of Singapore, Singapore, Singapore

**Keywords:** sub-lethal oxidative stress, lysosomal membrane permeabilization, caspase 3, transcription factor EB, lysosomes, Autophagy

## Abstract

Here we provide evidence to link sub-lethal oxidative stress to lysosome biogenesis. Exposure of cells to sub-lethal concentrations of exogenously added hydrogen peroxide resulted in cytosol to nuclear translocation of the Transcription Factor EB (TFEB), the master controller of lysosome biogenesis and function. Nuclear translocation of TFEB was dependent upon the activation of a cathepsin-caspase 3 signaling pathway, downstream of lysosomal membrane permeabilization and accompanied by a significant increase in lysosome numbers as well as induction of TFEB-dependent lysosome-associated genes expression such as *Ctsl*, *Lamp2* and its spliced variant *Lamp2a*, *Neu1*, *Ctsb*, *Sqstm1*, and *Atg9b*. The effects of sub-lethal oxidative stress on lysosomal gene expression and biogenesis were rescued upon gene silencing of caspase 3 and TFEB. Notably, caspase 3 activation was not associated with phenotypic hallmarks of apoptosis, evidenced by the absence of caspase 3 substrate cleavage, such as PARP, Lamin A/C or gelsolin. Taken together, these data demonstrate for the first time an unexpected and non-canonical role of a cathepsin-caspase 3 axis in the nuclear translocation of TFEB leading to lysosome biogenesis under conditions of sub-lethal oxidative stress.

## INTRODUCTION

Depending on the level of reactive oxygen species (ROS), cells elicit varied responses to oxidative stress, such as proliferation, growth arrest, apoptosis, autophagy or necrosis [1, 2]. An increase in intracellular ROS is also associated with cellular aging, which is attributed to the accumulation of damage to macromolecules upon chronic oxidative stress [3–5]. Contrarily, some authors have recently proposed that ROS may actually be involved in preventing cellular aging and promoting longevity [6, 7]. In this regard, autophagy has been proposed to be associated with the prevention of cellular aging [8, 9]. Autophagy is a catabolic mechanism that involves degradation and clearance of misfolded proteins and damaged organelles through the action of lysosomes and lysosomal hydrolases. Of note, an increase in autophagy is crucial in maintaining normal cellular homeostasis and survival upon exposure to sub-lethal oxidative stress [10, 11]. The proposed mechanisms involved in the activation of autophagy by ROS include inhibition of mTOR activity [12], activation of extracellular regulated kinase (ERK) and/or c-Jun N-terminal kinase [13, 14], nuclear translocation of the high-mobility group box 1 Protein, as shown upon the activation of autophagy by Apogossypolone [15], increase in Beclin 1 expression, and inhibition of the cysteine protease Atg4 activity [16]. However, while these mechanisms mainly focus on the regulation of the formation of autophagic vacuoles, ROS have also been shown to affect lysosomes [17]. Lysosomes and lysosomal proteases play important roles in the final stages of autophagy, such as the fusion of lysosomes with autophagosomes forming autolysosomes followed by proteolytic degradation of the engulfed molecules by lysosomal proteases [19]. Notably, lysosomal numbers, composition and function are sensitive to various external and internal stresses including redox stress [18]. For example, exposure of HeLa cells to H_2_O_2_, 6-hydroxydopamine (6-OHDA), and UVB irradiation led to an increase in lysosome numbers as well as lysosomal activity [17].

Lysosomes are ubiquitous membrane-bound intracellular organelles that are critical in pH-dependent macromolecule degradation, endocytic, heterophagy and autophagy pathways. In contrast to the rather simplified view of lysosomes as waste bags, lysosomes are now recognized as advanced organelles that mediate a variety of physiological processes crucial for the regulation of cell homeostasis, such as cellular clearance, lipid homeostasis and energy metabolism [20, 21]. A major player in the regulation of lysosomal biogenesis and function is the Transcription Factor EB (TFEB) [22]. TFEB induces the up-regulation of lysosomal genes including lysosomal cathepsins, lysosomal membrane proteins, and components of the vacuolar H+-ATPase [22, 23]. TFEB also regulates lysosome clearance by facilitating lysosome exocytosis [24]. Under basal conditions in most cell types, TFEB is located in the cytoplasm; however, under specific conditions, such as starvation or lysosomal dysfunction, TFEB rapidly translocates to the nucleus [25, 26]. This process is controlled by the phosphorylation status of TFEB; phosphorylated TFEB is located predominantly in the cytoplasm, whereas the dephosphorylated form is found in the nucleus [25, 26]. Despite the reported role of ROS on the physiology of lysosomes, the effects of sub-lethal oxidative stress on lysosome biogenesis and function have not yet been assessed. In the present report, we demonstrate that sub-lethal oxidative stress induces lysosome biogenesis and show for the first time an unexpected role for a cathepsin-caspase 3 axis in the activation of TFEB, the master controller of lysosome biogenesis and function.

## RESULTS

### Evidence of sub-lethal oxidative stress in L6 cells upon exposure to 50μM of exogenous H_2_O_2_

**Figure 1:**
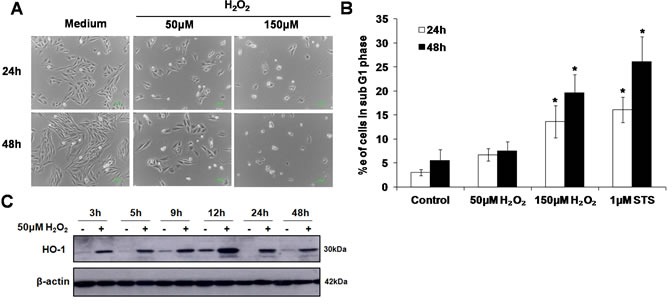
Sub-lethal oxidative stress does not induce apoptotic cell death **A**. L6 myoblasts were exposed to 50μM or 150μM of exogenous H_2_O_2_ for 24 and 48h before cell morphology was observed under a phase contrast microscope. **B**. L6 myoblasts were treated with 50μM or 150μM of exogenous H_2_O_2_, or 1μM STS, for 24h and 48h.Percentage of cells in sub-G1 phase was assessed using the propidium iodide (PI) staining, statistical analysis was done by comparing treatment (H_2_O_2_ or STS) to untreated control at respective time points (24h or 48h), values represent mean +/- SEM. *P < 0.05; *n* = 4 (*t*-test). **C**. HO-1 protein expression in L6 cells exposed to 50μM H_2_O_2_ for the indicated time points.

A concentration of H_2_O_2_ that induces oxidative stress in the absence of cell death was determined by exposing cells to 50μM or 150μM of exogenous H_2_O_2_. Results showed that incubation with 50μM of exogenous H_2_O_2_ neither significantly affected cell morphology (Figure 1A) nor resulted in an increase in the number of cells in the sub-G1 phase (marker of apoptosis) (Figure 1B). In contrast, cells exposed to 150μM H_2_O_2_ showed shrunken and rounded cell morphology (Figure 1A), and an increase in the number of cells in the sub-G1 phase that was comparable to the number of apoptotic cells detected following exposure to the classical inducer of apoptosis, staurosporine (STS) (Figure 1B). Interestingly, despite no evidence of untoward effects on cell morphology and survival, exposure to 50μM of exogenous H_2_O_2_ resulted in an increase in the expression of the stress-response protein HO-1, thereby indicating the induction of sub-lethal oxidative stress. The expression of HO-1 could be detected as early as 3h and sustained for at least 48h following the cells’ exposure to the oxidant (Figure 1C). Note that the increase in HO-1 expression in the control and H_2_O_2_ treated cells at 9h and 12h time points may reflect cell cycle- dependent expression of HO-1 [27] [28]. HO-1 expression was also detected in cells exposed to 150μM H_2_O_2_ but not in cells incubated with staurosporine (Supplementary Figure S1). Taken together these data support the use of 50μM H_2_O_2_ to assess the effect of a sub-lethal oxidative stress on lysosome biogenesis.

### Sub-lethal oxidative stress induces lysosome biogenesis

**Figure 2:**
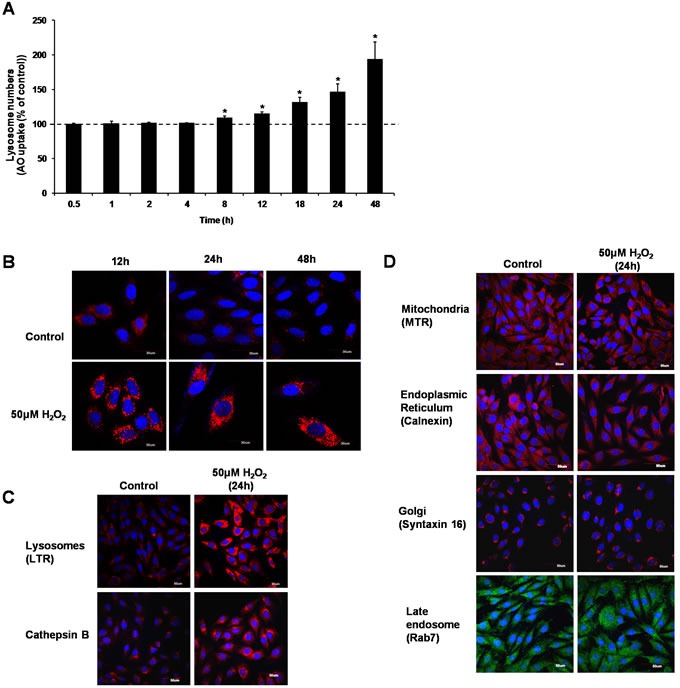
Sub-lethal oxidative stress induces lysosome biogenesis L6 cells were exposed to 50μM H_2_O_2_ for various time points before increase in lysosome numbers was assessed using **A**. AO uptake assay, values represent mean of the % of AO uptake compared to control cells at the same time point +/-SEM. *P < 0.05; *n* = 4 (*t*-test), or **B**. LTR uptake assay. Results are shown as confocal microscopy image showing cells with an increase in red fluorescence. Scale = 30μm. **C**. L6 cells were treated with 50μM H_2_O_2_ for 24h before being stained with LTR or with an antibody specific to Cathepsin B. Results are shown as confocal microscopy image showing cells with an increase in red fluorescence for LTR staining and for cathepsin B expression. Scale = 50μm. **D**. L6 cells were treated with 50μM H_2_O_2_ for 24h before being stained with organelle-specific antibodies or fluorescence dyes: mitochondria: MitoTracker Red CMXRos (MTR), endoplasmic reticulum: Calnexin, golgi: Syntaxin 16, late endosome: Rab7. Results are shown as confocal microscopy image. Scale = 50μm.

Acridine orange (AO), and LysoTracker® Red DND-99 (LTR) uptake assays were used to assess the effect of 50μM H_2_O_2_ on the lysosomal compartment. Cells were exposed to 50μM H_2_O_2_ before AO uptake and LTR staining were performed. Results showed a significant increase in the uptake of AO from 8h to 48h (Figure 2A) and LTR staining at 12h, 24h and 48h following cells’ exposure to H_2_O_2_ (Figure 2B). To confirm the effect of H_2_O_2_ on lysosomal compartment, expression of the lysosomal cysteine protease, cathepsin B, was determined using immunostaining in cells exposed to 50μM H_2_O_2_ for 24 hours. Figure 2C shows an increase in cathepsin B expression in cells exposed to sub-lethal oxidative stress. Finally, to rule out the effect of a sub-lethal oxidative stress on other cellular compartments, Mitotracker, and antibodies against calnexin, syntaxin 16 and Rab7 were used to assess the effect on mitochondria, endoplasmic reticulum (ER), the Golgi complex and late endosomes, respectively. Results showed no increase in mitochondria number or the expression of calnexin, syntaxin 16 and Rab7 proteins (Figure 2D). Taken together, these data support that sub-lethal oxidative stress specifically affects lysosome biogenesis.

### Sub-lethal oxidative stress activates TFEB

**Figure 3:**
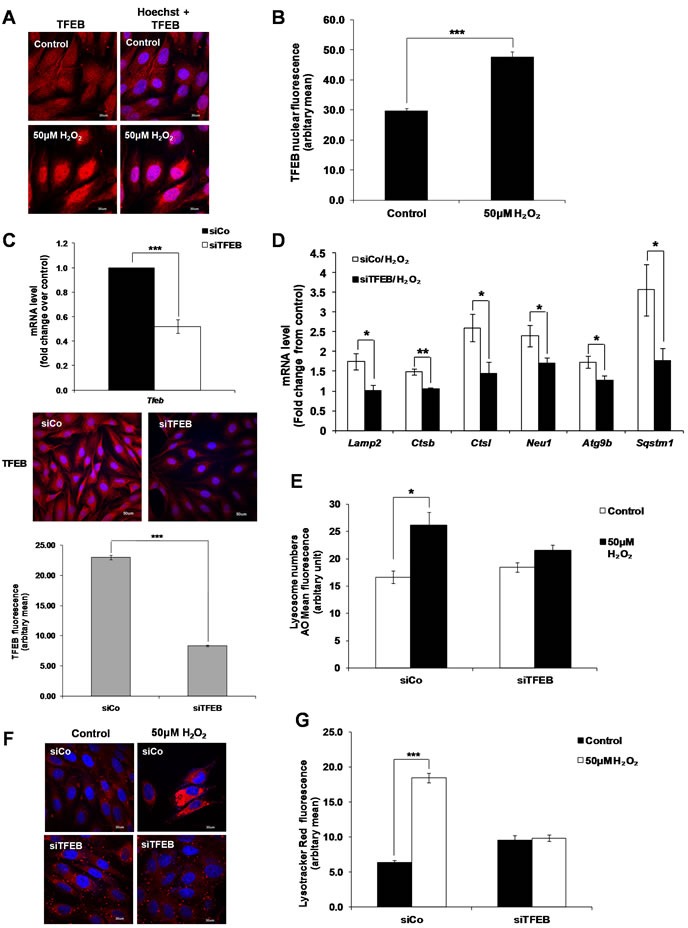
TFEB regulates lysosome biogenesis induced by a sub-lethal oxidative stress **A**. TFEB translocation from the cytosol to the nucleus following cells’ exposure to 50μM H_2_O_2_ for 24 Hours. Results are shown as confocal microscopy image showing a translocation of the red fluorescence from the cytosol to the nucleus. Scale = 30μm. **B**. Analysis of TFEB positive nucleus in control and treated cells from (A) quantified by ImageJ. Values represent mean +/- SEM. ***P < 0.0005; control: *n* = 122, treated: *n* = 78 (*t*-test). **C**. mRNA expression of TFEB was assessed in L6 myoblasts transfected with a siRNA specific to TFEB for 48h (siTFEB) using SYBR Green Real-Time PCR, normalized to endogenous control 18s. Relative mRNA expression is expressed as fold change over cells transfected with a control siRNA (siCo). Values represent mean +/- SEM. ***P < 0.0005; *n* = 11 (*t*-test). Images show TFEB fluorescence (red) in cells transfected with siCo and siTFEB for 48h. TFEB fluorescence staining in siCo and siTFEB cells was then quantified by ImageJ. Values represent mean +/- SEM, ***P < 0.0005; siCo: *n* = 144, siTFEB: *n* = 169 (t-test). **D**.-**F**. L6 cells were transfected with siTFEB or negative control siRNA (siCo) and exposed to 50μM H_2_O_2_. (D) mRNA expression of *Lamp2, Ctsb, Ctsl, Neu1, Atg9b and Sqstm1*, as quantified by SYBR Green Real-Time PCR and normalized to endogenous control 18s. Relative mRNA expression is expressed as fold change over untreated control. Values represent mean+/- SEM. *P < 0.05, **P < 0.005, *n* = at least 4 (t-test). Lysosome numbers were determined using (E) AO uptake assay or (F) LTR immunofluorescence assay. (E) Values represent AO mean fluorescence in arbitrary units +/- SEM,*P < 0.05; *n* = 4 (t-test). The inhibitory effect of siTFEB on the increase of AO mean of fluorescence was statistically significant *P <* 0.05 (mixed model). (F) Results are shown as confocal microscopy image showing cells with an increase in red fluorescence as in Figure 2B. Scale = 30μm. **G**. Analysis of the LTR immunofluorescence assay shown in (F) using ImageJ. Values represent mean +/- SEM, ***P < 0.005; sico/control: *n* = 66, sico/H_2_O_2_; *n* = 66, siTFEB/control: *n* = 59, siTFEB/H_2_O_2_: *n* = 85 (t-test). The effect of siTFEB was statistically significant, *P* < 0.05 (mixed model).

TFEB is the master regulator of lysosome biogenesis [22]. Overexpression of TFEB induces the expression of lysosomal genes, increases the number of lysosomes and promotes the ability of cells to degrade lysosomal substrates [22]. Although no significant change in TFEB mRNA expression was detected (Supplementary Figure S2), translocation of TFEB from the cytosol to the nucleus was observed in H_2_O_2_-treated cells (Figure 3A and 3B Supplementary Figure S13). Moreover, analysis of the mRNA expression of 18 lysosomal and autophagy-related TFEB target genes [22, 25, 29, 30] in untreated versus H_2_O_2_-treated cells showed that 4 genes associated with lysosome function (*Ctsl, Lamp2* and its spliced variant *Lamp2a, Neu1* and *Ctsb*) and 2 genes associated with autophagy (*Sqstm1* and *Atg9b*) were induced (at least 1.5 fold) by sub-lethal oxidative stress, compared to untreated cells (Table 1). Gene silencing of TFEB using a specific TFEB siRNA (Figure 3C and Supplementary Figure S13) significantly prevented the increase in mRNA expression of these 6 genes following cells’ exposure to sub-lethal oxidative stress (Figure 3D). Finally, a decrease in TFEB expression prevented the increase in lysosome numbers following cells’ exposure to 50μM of exogenous H_2_O_2_ (Figure 3E, 3F and 3G).

**Table 1:** Expression of selected TFEB gene targets following sub-lethal oxidative stress

*Gene symbol (Rat)*	*Fold increase+/-SEM*	*Protein function*
**Lysosomal genes**		
*Ctsl*	3.84 +/- 0.62	Lysosomal cysteine proteinase
*Neu1*	2.76 +/- 0.31	Lysosomal sialidase
*Lamp2*	2.34 +/- 0.28	Lysosomal membrane glycoprotein
*Ctsb*	1.63 +/- 0.17	Lysosomal cysteine protease
*Lamp2a*	1.47 +/- 0.12	Lysosomal receptor for Chaperone –Mediated Autophagy (CMA)
*Lamp1*	1.32 +/- 0.13	Lysosomal membrane glycoprotein
*Mcoln1*	1.27 +/- 0.18	Lysosomal cation channel
*Gba*	1.25 +/- 0.06	Lysosomal housekeeping enzyme
*Clcn7*	1.22 +/- 0.06	Lysosomal chloride channel
*Hexa*	1.05 +/- 0.17	Alpha subunit of the lysosomal enzyme β-hexosaminidase A
*Clcn3*	0.99 +/- 0.11	Lysosomal chloride channel
**Autophagy genes**		
*Sqstm1*	6.78 +/- 1.22	Ubiquitin and LC3 binding protein
*Atg9b*	2.14 +/- 0.21	Involved in autophagosome assembly and antisense transcript in the posttranscriptional regulation of eNOS3
*Map1c3b*	1.12 +/- 0.11	Ubiquitine like protein- involved in autophagosome formation
*Vsp11*	1.10 +/- 0.64	Involved in autophagosome and lysosme fusion
*Becn1*	0.96 +/- 0.08	Involved in autophagosomes formation, Bcl2 interacting protein
*Vps18*	0.85 +/- 0.07	Involved in autophagosome and lysosome fusion
*Bcl2*	0.83 +/- 0.15	Negatively regulate autophagy

Comparative ΔΔ*C*_t_ method was used to determine gene expression 24 hours following cells exposure to 50μM H_2_O_2_. Expression levels were normalized to the expression levels of the housekeeping gene 18s. Fold change compared to untreated cells represent the average of at least 3 independent experiments. Results are shown as fold increase +/- SEM.

### Sub-lethal oxidative stress induces an increase in the autophagic compartment in a TFEB-independent manner

**Figure 4:**
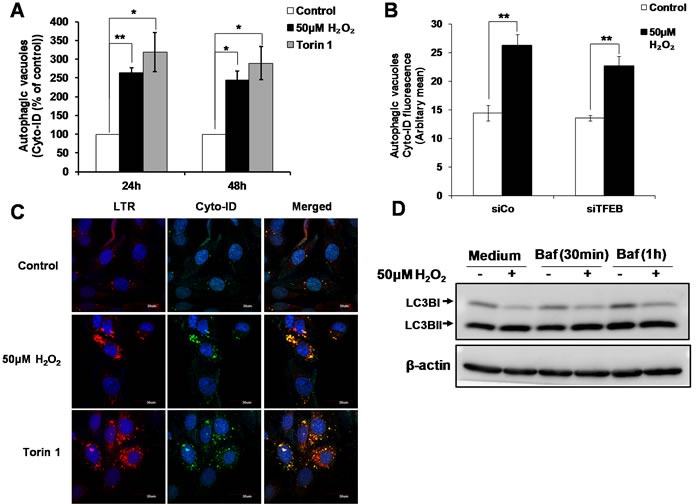
TFEB is not involved in the increase in autophagic vacuoles induced by sub-lethal oxidative stress **A**. Autophagic vacuoles in L6 cells exposed to 50μM H_2_O_2_ and 100nM Torin1 for 24h and 48h were detected using Cyto-ID staining, statistical analysis was done by comparing treatment (H_2_O_2_ or Torin1) to untreated control at respective time point (24h or 48h). Values represent mean +/- SEM, *P < 0.05, **P < 0.005; *n* = 4 (t-test). **B**. Detection of autophagic vacuoles in cells transfected with siTFEB or negative control siRNA (siCo), and exposed to 50μM H_2_O_2_ for 24h using Cyto-ID staining. Values represent mean +/- SEM, **P < 0.005; *n* = 5 (t-test). The effect of siTFEB was not statistically significant, *P*>0.05 (mixed model). **C**. Detection and co-localization of lysosomes and autophagic vacuoles using LTR and Cyto-ID respectively in L6 cells treated with 50μM H_2_O_2_ and 100nM Torin1 for 24h. Results are shown as confocal microscopy image. Scale = 30μm. **D**. L6 cells were treated with 50μM H_2_O_2_ for 24h. Cells were then incubated with 200nM Bafilomycin for 30min/1h before cells were harvested for LC3II Western Blot analysis.

Apart from being the master regulator of lysosome biogenesis, TFEB also controls autophagy by positively regulating autophagosome formation and autophagosome-lysosome fusion, both *in vitro* and *in vivo* [25]. Using the Cyto-ID autophagy detection assay, we observed an increase in the numbers of autophagic vesicles at 24 and 48h following the exposure of cells to 50μM H_2_O_2_. The increase in Cyto-ID staining induced by sub-lethal oxidative stress was comparable to that induced by Torin1, a classical inducer of autophagy (Figure 4A). However, while decreasing TFEB expression prevented lysosome biogenesis (Figure 3E, 3F and 3G), it did not significantly prevent the increase in Cyto-ID staining in cells exposed to sub-lethal concentration of H_2_O_2_ (Figure 4B). The effect of siTFEB on the increase in autophagic vacuoles assessed using a mixed model method as described in materials and methods was not statistically significant (*P*>0.05). This result suggests that the autophagic vacuoles formation upon cells’ exposure to a sub-lethal oxidative stress is independent of TFEB (Figure 4B). Moreover, although the co-localization of Cyto-ID and LTR (Figure 4C) and GFP-LC3 and LTR (Supplementary Figure S3) staining supported a fusion of lysosome and autophagosome leading to autolysosome formation, no autophagic flux could be demonstrated 24h following cells’ exposure to a sub-lethal oxidative stress (Figure 4D).

### Sub-lethal oxidative stress activates caspase 3

**Figure 5:**
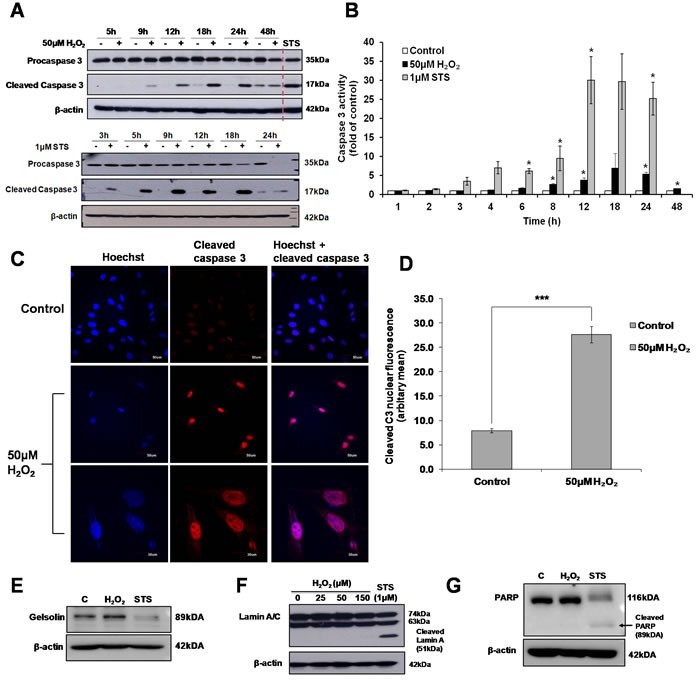
Sub-lethal oxidative stress induces the activation of caspase 3 L6 cells were exposed to 50μM H_2_O_2_ or 1μM STS for the indicated time points before **A**. Western Blot analysis of caspase 3 cleavage and **B**. Caspase 3 activity was assessed, statistical analysis was done by comparing treatment (H_2_O_2_ or STS) to untreated control at respective time point (24h or 48h). Values represent mean +/- SEM, *P < 0.05; *n* = 4 (t-test). **C**. Detection of cleaved caspase 3 by immunofluorescence analysis of cells exposed to 50μM H_2_O_2_ for 24h, as viewed under a confocal microscope with low magnification (scale = 50μm) and high magnification (scale = 30μm). **D**. Analysis of the nuclear localization of cleaved caspase 3 shown in (C). Values represent mean +/- SEM, ***P < 0.0005; control: *n* = 49, treated: *n* = 73 (t-test). **E**.-**G**. Western Blot analysis of the cleavage of classical caspase 3 substrate in cells exposed to 50μM H_2_O_2_ or 1μM STS: (E) Gelsolin, (F) Lamin A/C and (G) PARP.

Although exposure of cells to 50μM H_2_O_2_ was insufficient to induce apoptotic cell death (Figure 1A and 1B), activation of caspase 3 was observed in cells exposed to 50μM H_2_O_2_. Caspase 3 enzymatic activity and protein cleavage was time-dependent. Caspase 3 activity was significantly lower and detected at a later time point compared to the activation of caspase 3 induced in cells exposed to the apoptotic trigger STS (Figure 5A and 5B). Interestingly, unlike in STS-treated cells where cleaved caspase 3 relocated to the cytosol following a transient accumulation in the nucleus (Supplementary Figure S4), cleaved caspase 3 was retained in the nucleus in cells exposed to 50μM H_2_O_2_ (Figure 5C and 5D). Finally, the absence of the cleavage of classical apoptotic substrates such as the structural proteins, Gelsolin (Figure 5E), Lamin A/C (Figure 5F), and the DNA damage repair protein PARP (Figure 5G) in 50μM H_2_O_2_-treated cells concurs with the absence of apoptotic cell death in cells exposed to a sub-lethal oxidative stress.

### Sub-lethal oxidative stress induces caspase 3 activation in a cathepsin-dependent manner

**Figure 6:**
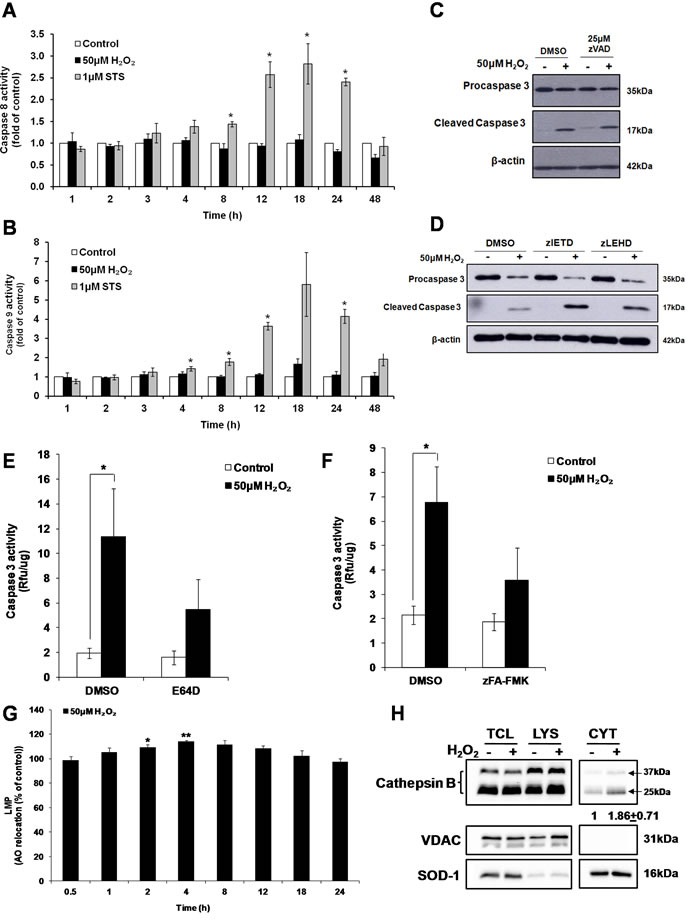
Cathepsin(s) induces the activation of caspase 3 by sub-lethal oxidative stress **A**. Caspase 8 and **B**. Caspase 9 activity in cells exposed to 50μM H_2_O_2_ or 1μM STS for indicated time points, statistical analysis was done by comparing treatment (H_2_O_2_ or STS) to untreated control at respective time point (24h or 48h). Values represent mean+/- SEM, *P < 0.05; *n* = 4 (t-test). **C**.-**D**. Western Blot analysis of caspase 3 cleavage by 50μM H_2_O_2_, in the presence of (C) pan caspase inhibitor (zVAD-FMK), or (D) caspase 8 (zIETD-FMK) or caspase 9 (zLEHD-FMK) inhibitor. **E**.-**F**. Prior to treatment with 50μM H_2_O_2_, cells were pre-treated with (E) E64D (50μM) or (F) zFA-FMK (50μM). Cell lysate collected was assayed for caspase 3 activity with the fluorogenic substrate Ac-DEVD-AFC. Values represent mean + /- SEM, *P < 0.05; *n* = 4 (t-test). The inhibitory effect of E64D and zFA-FMK on the increase in caspase 3 activity was statistically significant *P* < 0.05 (mixed model). **G**. LMP in cells treated with 50μM H_2_O_2_ for indicated time points, measured by AO relocation assay. Values represent mean of the % of AO relocation compared to control cells at the same time point, mean +/-SEM, *P < 0.05; *n* = 4 (t-test). **H**. L6 cells were treated with 50μM H_2_O_2_ for 4h and cathepsin B was blotted in different fractions. TCL: total cell lysate (35μg); LYS: lysosome/membrane (70μg); CYT: cytosol (20μg). Band intensity of cathepsin B was quantified after normalization to loading control. Changes in cathepsin B protein level in the cytosol fraction is shown as the fold difference relative to control cells. Values represent the means +/-SEM of two independent experiments.

While exposure to the classical apoptosis inducer STS resulted in the activation of the initiator caspases 8 and 9, no increase in caspase 8 and 9 activity were detected in cells exposed to sub-lethal oxidative stress (Figure 6A and 6B). Furthermore, the pan-caspase inhibitor zVAD-FMK (Figure 6C) or the tetra-peptide inhibitors of caspase 8 (zIETD-FMK) or caspase 9 (zLEHD-FMK) did not inhibit caspase 3 cleavage in H_2_O_2_-treated cells (Figure 6D). In order to determine the protease(s) involved in the activation of caspase 3, cells were pre-incubated with different protease inhibitors before the addition of 50μM H_2_O_2_. Inhibition of aspartate proteases using pepstatin A (Supplementary Figure S5A) or serine proteases by pefabloc (Supplementary Figure S5B) or calpains using calpeptin (Supplementary Figure S5C) did not significantly block the activation of caspase 3. On the contrary, the presence of the general cysteine protease inhibitor E64D (Figure 6E) and cathepsin B and L inhibitor zFA-FMK (Figure 6F) significantly inhibited the increase in caspase 3 activity. To eliminate the possibility that the effect of E64D or zFA-FMK inhibitors on caspase 3 activity might be due to a direct cross inhibition of caspase activity rather than the inhibition of an upstream cysteine protease involved in the cleavage of caspase 3, caspase 3 enzymatic activity was measured upon addition of the inhibitors to STS-treated cell lysate that contains fully activate caspase 3. No inhibition of caspase 3 activity by E64D (Supplementary Figure S6A) or zFA-FMK (Supplementary Figure S6B) was detected even at the concentration of the inhibitors as high as 100μM. In contrast, addition of 20μM of pan-caspase inhibitor, zVAD-FMK completely abrogated caspase 3 activity from the same STS-treated cell lysate (Supplementary Figure S6). Taken together these results support that cathepsins, in particular cathepsins B or/and L, are responsible for the activation of caspase 3 upon cells’ exposure to a sub-lethal concentrations of H_2_O_2_.

### Sub-lethal oxidative stress induces lysosomal membrane permeabilization

*In vitro* cleavage of caspases by cathepsins has previously been reported [31, 32]. In particular, two studies demonstrated the activation of caspase-3-like protease by digitonin-treated lysosomes and the participation of a cathepsin L-type protease in the activation of caspase-3 [33, 34]. As cathepsins are lysosomal proteases, while caspase 3 is a cytosolic protease, relocalization of cathepsins to the cytosol would be necessary to execute the processing of caspase 3. Release of cathepsins and other lysosomal hydrolases from the lysosome to the cytosol is shown to occur through a process called Lysosomal Membrane Permeabilization (LMP). To measure LMP, acridine orange (AO) relocation assay was used. Cells were pre-loaded with 10μM AO for 30min before being exposed to 50μM H_2_O_2_ and the LMP was measured at indicated time points. FACS analysis of AO fluorescence revealed a transient but significant (8% and 14%) increase in green fluorescence intensity at 2h and 4h respectively, following exposure of cells to H_2_O_2_ (Figure 6G). In addition, 4 hours following cells’ exposure to the sub-lethal oxidative stress and the time of maximum LMP, an increase in activated cathepsin B was detected using cell fractionation in the cytosol of cells exposed to 50μM H_2_O_2_ (Figure 6H)

### Inhibition of caspase 3 prevents the activation of TFEB upon sub-lethal oxidative stress

**Figure 7:**
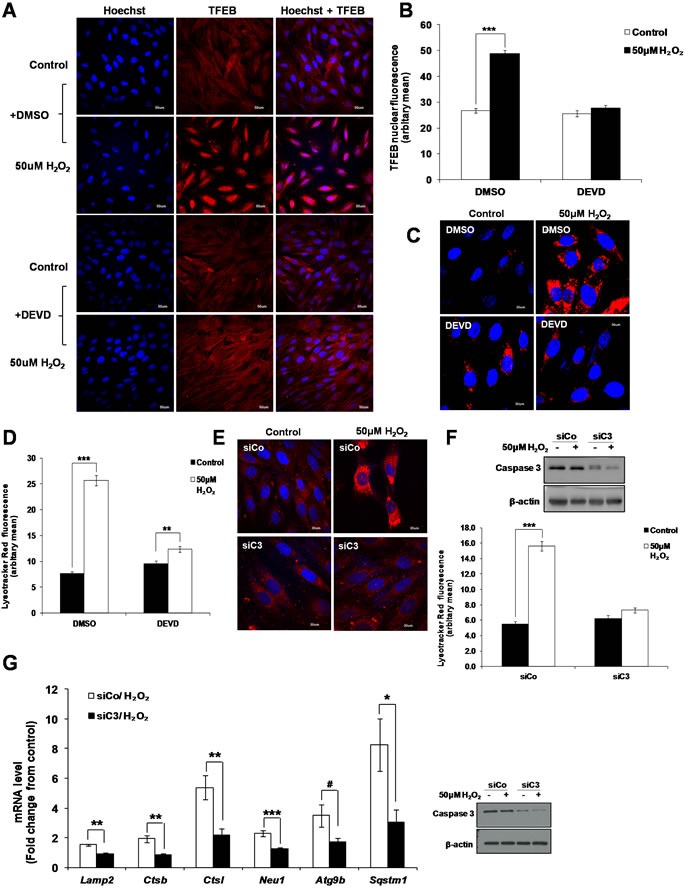
Caspase 3 is involved in the activation of TFEB leading to lysosome biogenesis **A**. L6 cells were pretreated with 20μM zDEVD-FMK for 2h before treatment with 50μM H_2_O_2_ for 24h and TFEB intracellular location was assessed using a TFEB specific antibody. Scale = 50μm. **B**. Analysis of the red nuclear fluorescence representing nuclear TFEB in control and treated cells shown in (A) quantified by ImageJ. Values represent mean +/- SEM, ***P < 0.0005; DMSO/control: *n* = 108, DMSO/H_2_O_2_: *n* = 103, DEVD/control: *n* = 103. DEVD/H_2_O_2_: *n* = 114 (t-test). The inhibitory effect of DEVD on the translocation of TFEB to the nucleus was statistically significant, *P* < 0.05 (mixed model). **C**.-**F**. Lysosome number was assessed using the LTR assay in (C) L6 cells pre-treated with 20μM zDEVD-FMK or (E) L6 cells transfected with a specific Caspase 3 siRNA (siC3) or negative control siRNA (siCo), before treatment with 50μM H_2_O_2_ for 24h. (D and F) Values represent mean +/- SEM, **P < 0.005, ***P < 0.005; (D) DMSO/control: *n* = 85, DMSO/H_2_O_2_: *n* = 78, DEVD/control: *n* = 78. DEVD/H_2_O_2_: *n* = 72 (t-test). (F) siCo/control: *n* = 69, siCo/H_2_O_2_: *n* = 95, siC3/control: *n* = 61. siC3/H_2_O_2_: *n* = 104 (t-test). The inhibitory effect of DEVD and siC3 on lysosome number were statistically significant *P* < 0.05 (mixed model). **G**. mRNA expression of TFEB target genes, *Lamp2*, *Ctsb*, *Ctsl*, *Neu1*, *Atg9b* and *Sqstm1*, as quantified by SYBR Green Real-Time PCR and normalized to endogenous control 18s in cells transfected with a specific Caspase 3 siRNA (siC3) or negative control siRNA (siCo). Relative mRNA expression is expressed as fold change over untreated control. Values represent mean +/- SEM,*P < 0.05, **P < 0.005, ***P < 0.0005, #*P* = 0.069; *n* = at least 3 independent experiments (t-test).

Having shown that sub-lethal oxidative stress concurrently induces lysosome biogenesis in a TFEB dependent manner and an LMP/cathepsin-dependent activation of caspase 3, we asked whether the two events could be associated. Indeed, inhibition of caspase 3 activity using the caspase 3 inhibitor zDEVD-FMK reduced the nuclear translocation of TFEB (Figure 7A and 7B). In addition, inhibition of caspase 3 activity using the zDEVD-FMK inhibitor (Figure 7C and 7D) or silencing of caspase 3 gene expression (Figure 7E and 7F, Supplementary Figure S8) prevented the increase in lysosome numbers induced by a sub-lethal oxidative stress and prevented the increase in the mRNA expression of the TFEB target genes *Lamp2, Ctsb, Ctsl, Neu1, Atg9b* and *Sqstm1* (Figure 7G).

### Chelation of iron prevents the activation of the caspase 3-TFEB-lysosome biogenesis by sub-lethal oxidative stress

**Figure 8:**
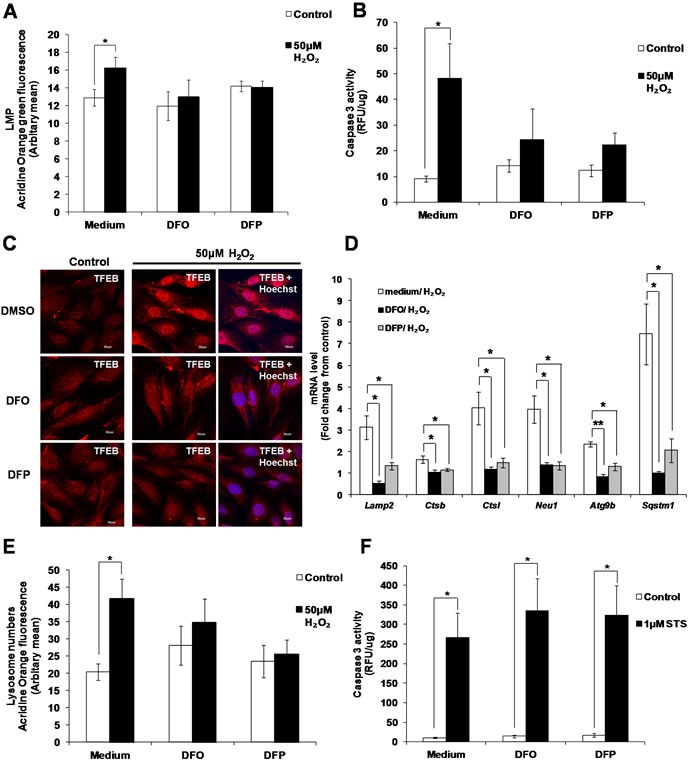
Activation of caspase 3 and lysosome biogenesis induced by subtoxic oxidative stress involves iron L6 cells were incubated with the iron chelators DFO (50μM) and DFP (50μM), before being exposed to 50μM H_2_O_2_. **A**. Values represent mean +/- SEM, *P < 0.05; *n* = 9 (t-test). The inhibitory effect of DFO and DFP on LMP was statistically significant, *P* < 0.05 (mixed model). **B**. Values represent mean +/- SEM, *P < 0.05; *n* = 5 (t-test). The inhibitory effect of DFO and DFP on caspase 3 activity was statistically significant, *P* < 0.05 (mixed model). **C**. TFEB cellular location in presence or absence of DFO and DFP (scale = 30μm), **D**. mRNA expression of TFEB target genes, *Lamp2*, *Ctsb*, *Ctsl*, *Neu1*, *Atg9b* and *Sqstm1*, and **E**. lysosome number in presence or absence of DFO and DFP was assessed as described in materials and methods. Values represent mean+/- SEM, *P < 0.05, **P < 0.005; *n* = at least 3 independent experiments (t-test). The inhibitory effect of DFO and DFP was statistically significant for *P* < 0.05 (mixed model). **F**. Caspase 3 activity in cells exposed to 1μM STS, pre-treated with DFO and DFP. Values represent mean+/- SEM, *P < 0.05; *n* = 5, (t-test). The effect of DFO and DFP was not statistically significant, *P*>0.05 (mixed model).

As the main site of organelle and protein degradation, lysosomes accumulate large amounts of free iron, which is susceptible to react with ROS during oxidative stress [39–41]. Reaction of iron with ROS in the lysosomes is associated with LMP [41–43]. Indeed, chelation of iron by 50μM Deferoxamine (DFO) and 50μM Deferiprone (DFP) prevented LMP induced by sub-lethal oxidative stress (Figure 8A). Inhibition of LMP upon iron chelation prevented the increase of caspase 3 activity (Figure 8B), the translocation of TFEB to the nucleus (Figure 8C), the increase in TFEB target genes expression *Lamp2, Ctsb, Ctsl, Neu1, Atg9b* and *Sqstm1* (Figure 8D), and lysosome biogenesis (Figure 8E) in cells exposed to 50μM H_2_O_2_. Finally, supporting that the pathway involved in the activation of caspase 3 by sub-lethal oxidative stress is different from the activation of caspase 3 upon the induction of apoptotic cell death, chelation of iron was unable to block STS-induced caspase 3 activation (Figure 8F).

### Activation of the caspase 3-TFEB-lysosome biogenesis pathway by a sublethal oxidative stress is not specific to the L6 cell lines

Having established an oxidative stress-dependent lysosome biogenesis pathway in L6 myoblasts involving caspase-3-mediated activation of TFEB in the absence of apoptotic cell death, we asked whether the same pathway could be activated in other cell lines. As shown in Supplementary Figure S9A, exposure of mouse embryonic fibroblast (MEF) or human fibroblast IMR90 cell line to sub-lethal oxidative stress, did not have any effect on cell morphology. Despite the absence of cell death, we observed the activation of caspase 3 which could be inhibited by the cathepsin inhibitor, zFA-FMK (Supplementary Figure S9B) and an increase in lysosomal gene expression upon exposure to sub-lethal concentrations of H_2_O_2_ (Supplementary Table S1; Supplementary Table S2). Lastly, inhibition of caspase 3 using zDEVD minimized the increase in lysosome biogenesis in MEF and IMR90 cells upon exposure to sub-lethal concentrations of H_2_O_2_ (Supplementary Figure S9C).

## DISCUSSION

Our study demonstrates that sub-lethal oxidative stress induces lysosome biogenesis through activation of TFEB in a caspase 3-dependent manner. Activation of the caspase cascade has long been a hallmark of apoptosis. In particular, biochemical and morphological changes during apoptosis, such as cell shrinkage and membrane blebbing [44], DNA fragmentation [45], and nuclear condensation [46] are manifestations of caspase 3 substrates cleavage. Nevertheless, in recent years, there have been increasing evidence of caspases activation and cleavage of their substrates in the absence of apoptotic cell death. The involvement of caspases in the absence of cell death has been demonstrated in developmental processes such as erythropoiesis and spermatid maturation [47–50]. Furthermore, caspases play important roles in differentiation of various cell types, such as osteogenic differentiation of bone marrow stromal stem cells [51], neuronal differentiation of primary derived neuronal stem cells [52], and in proliferation and differentiation of adult hematopoietic stem cells [53]. Recent findings from our lab also revealed a role of caspase 3 in regulating gene expression during oxidative stress. Caspase 3 and 6 were shown to regulate the expression of the Na+/H+ exchanger (NHE1) during oxidative stress in an iron-dependent pathway [54]. In the present study, we demonstrate yet another function of caspase 3 upon exposure of cells to sub-lethal oxidative stress.

Compared to their role in apoptotic cell death, the signaling pathway(s) activated by caspases in the absence of cell death are less well studied. The classical pathways leading to the activation of caspase 3 involve the activation of initiator caspase 8 and/or caspase 9. In the present report, caspase 8 and caspase 9 were not activated and inhibition of either of the protease did not affect the activation of caspase 3. However, cathepsins were shown to be involved in the activation of caspase 3. Although we do not identify which cathepsin(s) was/were directly involved in the activation of caspase 3, the result obtained using the cathepsins inhibitor zFA suggest that it may be cathepsin B or L. Direct cleavage of caspase 3 by cathepsins was previously reported *in vitro* [33, 34] and *in vivo* [55]. In order to activate caspase 3, active cathepsins need to translocate from the lysosome to the cytosol, a process that involves the induction of LMP. Indeed, LMP was observed 2h and 4h following the exposure of L6 to sub-lethal oxidative stress. The apparent transient nature of the LMP suggests that it was different from the lysosome rupture seen during necrotic and apoptotic cell death, which often results in massive release of lysosomal proteins and digestion of cellular proteins. Interestingly, it has previously been reported that in cases where cathepsins were detected in the cytosol, lysosomes could still be labelled with lysosomotropic fluorochromes, indicating that the cytosol-lysosome pH gradient was maintained [56] and that LMP may occur selectively in a subset of lysosomes [43]. Hence, the apparent transient LMP could represent an LMP in a subset of lysosomes, 2 and 4 hours following exposure to a sub-lethal oxidative stress. The limited amount of cathepsin relocated in the cytosol would then allow for a controlled activation of caspase 3 that will not allow the activation of apoptotic cell death.

The actual mechanism of LMP is largely unknown although ROS is reported to be one of the common mediators [57, 58]. Lysosome membranes are susceptible to oxidative stress due to the pool of labile, redox active intra-lysosomal iron, produced by the degradation of metalloproteins [59]. Exogenously-added H_2_O_2_ diffuses readily across the lysosome membranes and reacts with intra-lysosomal iron to form other ROS, such as ·OH and OH-, through the Fenton reaction [60]. Such formation of ROS may result in peroxidation of the lysosome membranes and thus induce LMP [61]. In the present study, chelating iron prevented the selective LMP and the activation of the caspase 3-dependent lysosome biogenesis pathway.

Several reviews have elegantly summarized the involvement of caspases in the absence of cell death, which point to mechanisms acting singularly or in combination for restricted cleavage of substrates and limited execution of apoptosis [62, 63]. In the present report, moderate activation of caspase 3 due to a limited cytosolic relocation of cathepsin(s) combined with the retention of cleaved caspase 3 in the nucleus may account for restricted substrate cleavage and absence of the apoptotic phenotype in cells exposed to sub-lethal oxidative stress.

While regulation of lysosome biogenesis by caspase 3 via TFEB is not reported in the literature, it was reported previously that a decrease in mTOR activity accompanied by its de-phosphorylation is associated with the activation of TFEB. In agreement with the effect of a redox stress on mTOR [12] we observed a de-phosphorylation of mTOR (Supplementary Figure S10) similar to the de-phosphorylation observed upon cells’ exposure to Torin. However, knock-down of caspase 3 protein expression did not prevent the decrease in phosphorylated mTOR following cells’ exposure to a sub-lethal oxidative stress (Supplementary Figure S11). These data support that the cathepsin(s)-caspase 3 dependent translocation of TFEB from the cytosol to the nucleus is a novel pathway independent of mTOR.

Activation of caspase 3 has been shown to inhibit autophagy via cleavage of ATG-related genes such as beclin-1 [64]. On the other hand, Sirois et al. reported that caspase 3 regulates the formation of the autophagic network in nutrient-deprived endothelial cells by controlling the maturation and release of autophagic vacuoles, although it has no effect on the autophagic flux [65]. Similarly, our results show the formation of autophagic vacuoles without the detection of autophagic flux. Moreover, contrary to the studies in which TFEB is reported as a master regulator of autophagy, our results support that upon cells’ exposure to sub-lethal level of H_2_O_2_, TFEB takes part in lysosome biogenesis but not in the autophagic vacuoles formation. Although we observed an increase in autophagic vacuoles, this was independent of TFEB's transcription activity. Notably, in most studies showing the effect of TFEB on autophagy, TFEB was overexpressed. In cells exposed to sub-lethal oxidative stress, TFEB nuclear translocation is induced without an increase in TFEB expression. Therefore, the expression level of TFEB may be important to determine the subsets of genes activated and the cellular process they activate. In support of this hypothesis, among the set of 18 known TFEB target genes assessed for their increase in transcription following cells’ exposure to a sub-lethal oxidative stress, only a subset of genes was activated. Of note, an increase in the *Lamp2* splicing form *Lamp2a* and *Sqstm1* mRNA was detected. Increase in Lamp2a and p62 (Sqstm1) proteins have both been associated with chaperone-mediated autophagy [66] and selective autophagy [67, 68]. Hence, we propose that sub-lethal oxidative stress might induce selective rather than macroautophagy via an increase in lysosome numbers in a caspase 3/TFEB-dependent pathway.

**Figure 9:**
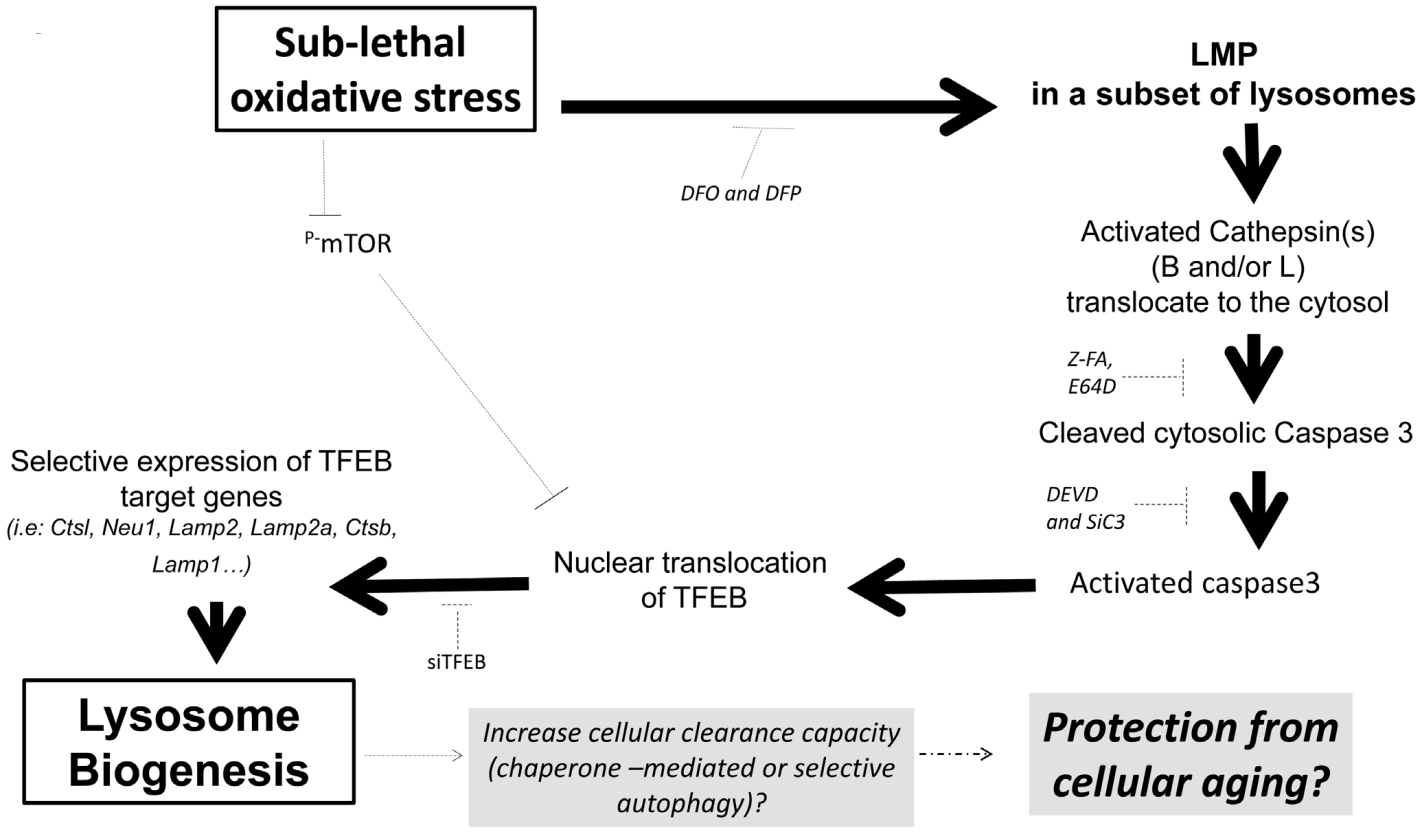
Sublethal oxidative stress activates an mTOR-independent signaling pathway involving the activation of a LMP-cathepsin-caspase 3 axis leading to the transcription of TFEB-target genes involved in lysosome biogenesis We propose that caspase 3-dependent activation of TFEB might be a protective mechanism from cellular aging through activation of chaperone-mediated or selective autophagy following an increase in lysosome biogenesis in absence of autophagic flux.

In conclusion, our report demonstrates a role for an LMP-cathepsin-caspase 3-TFEB axis in the regulation of lysosome biogenesis during sub-lethal oxidative stress. We propose that activation of the LMP-cathepsin(s)-caspase 3-TFEB axis could be associated with the acquisition of a lysosome-dependent clearance phenotype that may support the pro-survival effect of a sub-lethal oxidative stress. It should be pointed out that our data did not support the involvement of an autophagic flux while the expression of *Lamp2* was increased. Hence, activation of a LMP-cathepsin-caspase 3-TFEB axis leading to lysosome biogenesis may represent a novel signaling pathway that contributes to an increase in selective and/or chaperone-mediated autophagy rather than macroautophagy and plays a role in the increase in organism life span induced by low level of ROS (Figure 9). Finally, we also demonstrate that the LMP-cathepsin(s)- caspase 3 axis was also activated using etoposide (Supplementary Figure S12).

## MATERIALS AND METHODS

### Cell lines and cultures

L6 rat myoblasts were obtained from Dr. Larry Fliegel (Department of Biochemistry, University of Alberta, Canada). L6 cells were maintained in Dulbecco's modified Eagle's medium (DMEM) (Hyclone, SH30022.01) supplemented with 10% FBS (Hyclone, SV30160.03), 2mM L-glutamine (Hyclone, SH3003401), 0.25mg/ml Geneticin (Life Technologies, 11811-031), and 1mM Gentamicin Sulfate (Lonza, 17-519L) at 37°C, with 5% CO_2_ in a humidified atmosphere. MEF cells and IMR90 cells were maintained in DMEM supplemented with 10% FBS, 2mM L-glutamine and 1mM Gentamicin Sulfateat 37°C, with 5% CO_2_ in a humidified atmosphere.

### Reagents and chemicals

Hydrogen peroxide (H_2_O_2_) was purchased from Merck (107209). E64D was purchased from Calbiochem (330005). Pepstatin A (P5318), Deferoxaminemesylate salt (DFO) (D9533), Staurosporine (STS) (S5921), Pefabloc (76307) were obtained from Sigma, zVAD-FMK (FMK001), zDEVD-FMK (FMK004), zIETD-FMK (FMK007), zLEHD-FMK (FMK008) and zFA-FMK (FMKC01) were purchased from R&D system. Caspase 3 substrate (ALX-260-032), caspase 8 substrate (ALX-260-110) and caspase 9 substrate (ALX-260-116) were obtained from Enzo Life sciences. Deferiprone (DFP) (Acros, 278740050) was purchased from Acros Organic. Caspase 3 (9662), cleaved caspase 3 (9664) and Lamin A/C (4777) antibodies were purchased from Cell Signalling.Cathepsin B (ab33538) and cleaved caspase 3 (for immunofluorescence) antibody (ab13847) was purchased from Abcam. Mouse monoclonal Anti-β-actin antibody (A5441) was purchased from Sigma. Calpeptin (sc-202516), HO-1 antibody (sc-10789), TFEB antibody (sc-48784) and Gelsolin antibody (sc-48769) were purchased from Santa Cruz. PARP antibody (630210) was purchased from Clontech.

### Morphology studies

Cells morphology was observed under Nikon Eclipse TS100. Morphology pictures were taken with Nikon DS-Fi1c at the magnification of 10X.

### Caspase activity assay

Cell lysate were obtained with 1X Cell Lysis Buffer (BD Biosciences Pharmingen, 559759). 40μl cell lysate was added to 44μl of reaction mixture consisting of: 4μl of specific caspase substrate [1mM (stock conc)] and 40μl of 2X Reaction Buffer (10mM HEPES, pH 7.4, 2mM EDTA, 6mM DTT, 10mM KCl and 1.5mM MgCl_2_) supplemented with protease inhibitors (1mM PMSF (Sigma,78830), 10μg/ml aprotinin (Sigma, A3428), 10μg/ml pepstatin A (Sigma, P5318), 20μg/ml leupeptin (Sigma, L2884)). Samples were incubated at 37°C for 1h and fluorescence was read at an excitation wavelength of 400 nm and an emission wavelength of 505 nm using Spectrofluoro Plus spectroflurometer (TECAN). Caspase activity was normalized against protein concentration of each sample and expressed as relative fluorescence unit per microgram of protein (RFU/μg).

### Determination of the sub-G1 population

Cells were harvested, washed twice with 1XPBS, and resuspended in ice-cold PBS/1%FBS. Cells were then fixed with 70% ethanol at 4°C for 30min. After fixation, cell pellet was washed twice with ice-cold 1% FBS/PBS before incubation with 500μl of PI/RNaseA staining solution for 30min at 37°C in dark. Stained cell samples were analyzed by flow cytometry using PI Channel on the flow cytometer (BD FACSCanto II, BD Biosciences). Flow cytometry data was analyzed with CyflogicTM software (CyFlo Ltd).

### SDS-PAGE and immunoblotting

Cell lysates were prepared in RIPA lysis buffer containing 20 mm Tris (pH 7.5), 150mM NaCl, 1 mm EDTA, 1mM EGTA, 1% Triton X-100, supplemented with 1mM Na_3_VO_4_(Sigma, S6508), 1μg/ml leupeptin, 1μg/ml pepstatin A, 1μg/ml aprotinin and 1mM PMSF. Cell lysate were resolved by SDS-PAGE and probed with protein of interest using SuperSignal Chemiluminescent Substrate (Thermo Scientific, 34080) with Kodak Biomax MR X-ray film or BioradChemiDoc™ MP System.

### Analysis of cathepsin B localization with cellular fractionation

To analyze cathepsin B translocation after H_2_O_2_ treatment, lysosomal/membrane-cytosolic fractionation was performed as described as follows. Cells were washed twice with 1xPBS and incubated with MSH-Buffer (210 mM mannitol, 70 mM sucrose, 20 mM HEPES pH 7.5, 1 mM EDTA, 300 μM Pefabloc, 100 μM PMSF) for 45 min on ice. Cells were lysed with a 23-G needle and centrifuged for 5 min at 700g to attain pellet consisting of nuclei and cellular debris. The supernatant was centrifuged at 100,000g for 45min to separate the cytosol (supernatant) and the membrane/lysosomal fraction (the resulting pellet, which contains all other organelles except the nuclei, was resuspended in MSH buffer + 1% triton). The cell lysate (cytosolic and membrane/lysosomal fraction) obtained was resolved by SDS-PAGE and protein of interest was detected using SuperSignal Chemiluminescent Substrate (Thermo Scientific, 34080) with Biorad ChemiDoc™ MP System.

### RNA interference (RNAi) assay

Cellular transfection of siRNA was performed using Lipofectamine® RNAiMAX (Invitrogen, 13778150) in Opti-MEM®I reduced serum medium (Invitrogen, 31985070) according to the manufacturer's protocol. siRNA for caspase 3 (ON-TARGETplusSMARTpool - Rat CASP3), cathepsin B (ON-TARGETplusSMARTpool - Rat CTSB) and TFEB (ON-TARGETplusSMARTpool - Rat TFEB) were purchased from Dharmacon (Thermo Scientific). A negative control siRNA (QIAGEN, 1027423) that is non-homologous to any known gene sequence was used as a negative control.

### Immunofluorescence assay using confocal microscopy

Cells were seeded on coverslips and were fixed with 4% paraformaldehyde for 30min at room temperature. After permeabilization with 0.2% TX-100 (Sigma, X100) for 10min at room temperature, cells were incubated with cleaved caspase 3 antibody (ab13847), cathepsin B antibody (ab33538) or TFEB antibody (sc-48784) for 2h at room temperature. After washing with PBS, cells were incubated with Rhodamine RedTM-X goat anti-rabbit IgG (Molecular Probes, R6394) and with Hoechst 34580 (Molecular Probes, H21486) for 1h. Immunofluorescence images were viewed under Olympus FluoView1000 (FV1000; Olympus) with identical acquisition parameters for the same image session and analyzed with Olympus FLUOVIEW Ver1.7a Viewer. Cleaved caspase 3 and TFEB nuclear staining were quantified with the ImageJ software. Briefly, nucleus of cell of interest was defined using the drawing tool. Mean fluorescence of the nuclear staining was obtained by selecting the “measure” option. After deducting any background staining, the mean fluorescence of at least 50 cells from each treatment was used for statistical analysis. Data is represented as mean +/- SEM (standard error of mean).

### RNA isolation, reverse transcription and real-time PCR

Total RNA was isolated using RNeasy Mini Kit (QIAGEN, 74104) according to the manufacturer's instructions. Reverse transcription was performed using the TaqMan® Reverse Transcription Reagents kit (Life technologies, N8080234). Each RT reaction contains 2.5μg of total RNA, 1 X RT buffer, 5 mM MgCl_2_, 425μM each of dNTPs, 2μM random hexamers, 0.35 U/μl RNase inhibitor, 1.1 U/μl MultiScribe™ reverse transcriptase added up to total volume of 10μl with RNAse-free water. Real-time quantitative PCR reaction was carried out with SYBRGreen (Applied Biosystems, 4309155), detection using ABI PRISM 7300 (Applied Biosystems). The sequences of the primers used for PCR wereas follows: rat TFEB (FP:GGGCTACATCAACCCCGAAA, RP:GTCATTGGCCTTGGGGATCA), rat LAMP1 (FP:CACGACTGTGACCAGAGCAT, RP:GTGCTGAACGTGGGCTCTAT), rat LAMP2 (FP:AACCCTGCCACAACCAACTT, RP:GTATGATGGCGCTTGAGACC), rat LAMP2A (FP: GTCTCAAGCGCCATCATACT, RP: TCCAAGGAGTCTGTCTTAAGTAGC), rat CTSB (FP:TGAGGACCTGCTTACCTGCT, RP:GTAGCCAGCCTCACACATCT), rat CTSL (FP:TGTGCGCAGCTAGCCACCTC, RP:GCCGTGCTTCCCGTTGCTGT), rat CLCN3 (FP:CAAAGCCGGGTAGCAGTGAA, RP: GTAGCTGGCTGCTTATCTTGC), rat CLCN7 (FP:CGTGAGGATGACCCTTAGCC, RP:GGCGTGCTCATTACTTCCCT), rat GBA (FP:TCACCCACTTGGCTCAAGAC, RP:TTGGAAGGGGTACCCAGTGA), rat HEXA (FP:GCCCCAGTACATCCAAACCT, RP:GCTGTGACGACAGAGACCAT), rat MCOLN1 (FP:AAACACCCCAGTGTCTCCAG, RP:ACCAGCCATTGACAAACTCC), rat SQSTM1/p62 (FP:CTCAGCCCTCTAGGCATCG, RP:CCCTTCCGATTCTGGCATCT), rat ATG9B (FP:AGCCGTCTTGGTCAAGTGAT, RP:CCACCCATCCAATTTCCTGC), rat BCL2 (FP:GGATAACGGAGGCTGGGATG, RP:CGTCTTCAGAGACAGCCAGG), rat BECN1 (FP:CGTCGGGGCCTAAAGAATGG, RP:GAATGGTCACTCGGTCCAGG), rat MP1LC3B (FP:CGGGTTGAGGAGACACACAA, RP:TCTTTGTTCGAAGCTCCGGC), rat VPS11 (FP:CTGGTCTTTGGAGATATCCTTCCT, RP:TCCAGATCTTTACCAGGGGGTTA), rat VPS18 (FP:ATTGACTTCACCCCCTCCGA, RP:CTTGCCCAAGTCAATGCGGA), rat 18s (FP:CATTCGAACGTCTGCCCT, RP:GTTTCTCAGGCTCCCTCTCC), mouse LAMP1 (FP:AGCATACCGGTGTGTCAGTG, RP:GTTGGGGAAGGTCCATCCTG), mouse LAMP2 (FP:TGCTTTCTGTGTCTAGAGCGT, RP:CCTGAAAGACCAGCACCAACT), mouse CTSB (FP:TGTGGTGGTCCTTGATCCTT, RP:AATCTGTCCAATGGTCGGGC), mouse CTSL (FP:TCAGGGTGACATGGTACAGC, RP:CTAGTGGGGCTGGCAAGATA), mouse GBA (FP:GAGTGAATGAATGCAGGACGC, RP:GACACTCAGCTCCATCCGAC), mouse HEXA (FP:CAGAGCTCACCAGAAAGGGG, RP:GTTCACCGGTCCAAATGTGC), mouse MCOLN1 (FP:TTGGGCCAATGGATCAGCTT, RP:GTTCTTGTAACTGGCGCTGC), mouse NEU1 (FP:TTTGGAGTAAGGACGACGGC, RP:TGCAGCGGCAATGGTAGTTA), mouse SQSTM1/P62 (FP:GCTGAAGGAAGCTGCCCTAT, RP:TTGGTCTGTAGGAGCCTGGT), mouse ATG9B (FP:ACCTGTTCACTCAAGCGCAA, RP:CAGATGCCCAACCCAAACCT), human LAMP1 (FP:ACGTTACAGCGTCCAGCTCAT, RP:CCTGGGTGCCACTAACACAT), human LAMP2 (FP:GCCGTTCTCACACTGCTCTA, RP:CCGCTATGGGCACAAGGAA), human CTSB (FP:AGTGGAGAATGGCACACCCTA, RP:GTACTGATCGGTGCGTGGAA), human CTSL (FP:GAACCCAGACCCGAGGTTTT, RP:CAGCAGAGTTCGGGGTAGAC), human GBA (FP:TGGGTACCCGGATGATGTTA, RP:AGATGCTGCTGCTCTCAACA), human HEXA (FP:CGTTTGTCACACTTCCGCTG, RP:CCATTCACCTACAGCCAGCA), human MCOLN1 (FP:TCTTCCAGCACGGAGACAAC, RP:GCCACATGAACCCCACAAAC), human NEU1 (FP:GCACATCCAGAGTTCCGAGT, RP:CAGGGTTGCCAGGGATGAAT), human SQSTM1/P62 (FP:AAGGCCTACCTTCTGGGCAAG, RP:GTCACTGGAAAAGGCAACCAA), human ATG9B (FP:CACCTTGGGGTTGTCAGGTT, RP:GAGAAGAGCCTTCCCAAGCG) and human/mouse 18s (FP:GTAACCCGTTGAACCCCATT, RP:CCATCCAATCGGTAGTAGCG). Comparative ΔΔ*C*_t_ method was used to determine gene expression. Expression levels were normalized to the expression levels of the housekeeping gene 18s.

### Detection of autophagic vacuoles

For measurement of total autophagic vacuoles, cells were stained with Cyto-ID (Enzo Life Sciences, ENZ-51031-K200) according to manufacturer's instruction. For flow cytometry, cells were trypsinized and were incubated with Cyto-ID reagent for 30min at 37°C in the dark. Cells were then washed with 1XPBS and resuspended in 500μl PBS/FBS and were subjected to flow cytometry analysis using Alexa-488A channel on the flow cytometer (BD FACSCanto II). Flow cytometry data was analyzed with CyflogicTM software (CyFlo Ltd). For immunofluorescence, cells were incubated with Cyto-ID reagent for 30min at 37°C in dark. Cells were fixed with 4% parafomaldehyde and mounted onto glass slide for confocal microscopy analysis using a FITC filter. Fusion of autophagosomes and lysosomes was visualized by GFP-LC3 and LTR immunofluoresnce analysis. GFP-LC3 plasmid was obtained from Dr Liu Dan (Department of Physiology, National University of Singapore Prof S. Pervaiz Lab). Cells were transiently transfected with the GFP-LC3 plasmid using Lipofectamine 2000 (ThermoFisher Scientific, 1168-019), according to the manufacturer's instruction. Cells were then treated with 50μM H_2_O_2_ 24h after transfection. At 24h, cells were stained with Lysotracker® Red DND-99 (LTR) (Molecular Probes, L-7528) for 45min. Cells were fixed with 4% paraformaldehyde for 30min at room temperature and cover slips were mounted for confocal analysis using Olympus FluoView1000 (FV1000; Olympus) confocal microscope.

### Analysis of lysosomal membrane permeabilization with the acridine orange relocation assay

Cells were incubated with 10μM Acridine Orange (AO) (Molecular Probes, A1301) for 30min at 37°C before treatment with H_2_O_2_. AO cytosolic staining (Green fluorescence) was analyzed by flow cytometry using the FITC-A channel (BD FACSCanto II). Results are expressed as the mean +/- SEM of arbitrary fluorescence of each treatment of at least 3 independent experiments.

### Analysis of lysosome numbers with LysotrackerRed and acridine orange staining

Lysosomal numbers were assessed by immunofluorescence with LTR or by flow cytometry with AO staining. For immunofluorescence, cells seeded on cover slips were stained with LTR for 45 min, 37°C. Cells were then fixed with 4% paraformaldehyde for 30min at room temperature and cover slips were mounted for confocal analysis using Olympus FluoView1000 confocal microscope with identical acquisition parameters for the same image session. Quantification of LTR staining was done by ImageJ software. Cell of interest was defined using the drawing tool. Mean fluorescence of the LTR staining was obtained by selecting the “measure” option. After deducting background staining, the mean fluorescence of at least 50 cells from each treatment was used for statistical analysis. Data is represented as mean +/- SEM. For flow cytometric analysis of lysosome numbers, cells were exposed to a solution of 10μM AO for 30min after treatment with H_2_O_2_. AO staining (red fluorescence) were analyzed by flow cytometryusing the PerCP-A channel (BD FACSCanto II). Flow cytometry data were analyzed with CyflogicTM software. Results are expressed as the mean +/- SEM of arbitrary fluorescence of each treatment of at least 3 independent experiments.

### Statistical analysis and data representation

Mixed model was utilized to evaluate the effect of cells exposure to H_2_O_2_ in control medium versus cells exposed to H_2_O_2_ in presence of inhibitors or following gene silencing on different outcomes such as lysosome number, autophagic vacuole formation, and caspase 3 activity with the consideration that the correlation of the outcomes was obtained from the same experiment setting or same plate. Compound symmetric structure was chosen to model the variance-covariance matrix. A full factorial model, which included 2 main effects and 1 interaction, was used to assess the effect of H_2_O_2_ upon the presence of inhibitor or the silencing of a gene. Furthermore, a subgroup analysis was carried out to compare the effect of H_2_O_2_ on the outcome in presence of various inhibitors or upon silencing of different genes. Comparative ΔΔ*C*_t_ method was used to determine gene expression 24 hours following cells exposure to 50M H_2_O_2_. Expression levels were normalized to the expression levels of the housekeeping gene 18s. All Western Blot and immunofluorescence images are representative of at least three independent experiments. Two-tailed unpaired Student's t-test was performed when appropriate; using the Microsoft Excel software, with p-value (*P*) of less than 0.05 considered significant. All numerical data including error bars represent the mean +/- SEM (Standard error of mean).

## SUPPLEMENTARY MATERIAL FIGURES AND TABLES


