# Supplementary material for: Sub-lethal oxidative stress induces lysosome biogenesis via a lysosomal membrane permeabilization-cathepsin-caspase 3-transcription factor EB-dependent pathway
Source: Oncotarget. 2016 Dec 18;8(10):16170–89. doi: 10.18632/oncotarget.14016 (PMC5369955; doi:10.18632/oncotarget.14016)
Supplement: Supplementary file 1 [file oncotarget-08-16170-s001.pdf]

Sub-lethal oxidative stress induces lysosome biogenesis via a lysosomal membrane permeabilization-cathepsin-caspase 3-transcription factor EB-dependent pathway

Supplementary Material

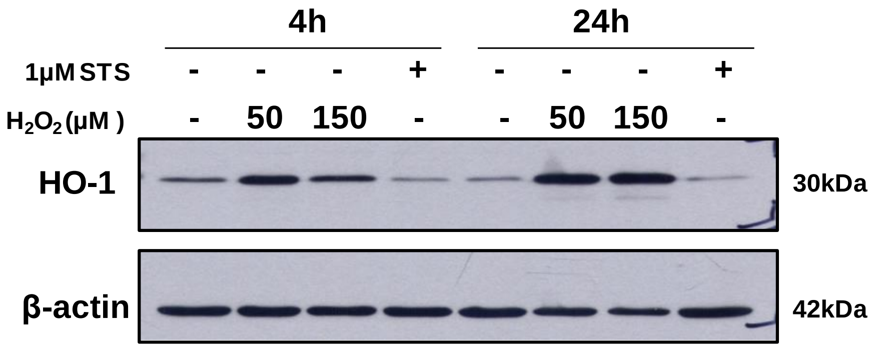

Supplementary Figure S1: H_2O_2 treatment induced the expression of the oxidative stress marker Heme Oxygenase 1 (HO-1). L6 cells were treated with 50µM H_2O_2, 150µM H_2O_2, or 1µM STS for 4h and 24h. Cell lysate was subjected to Western Blot analysis of HO-1.

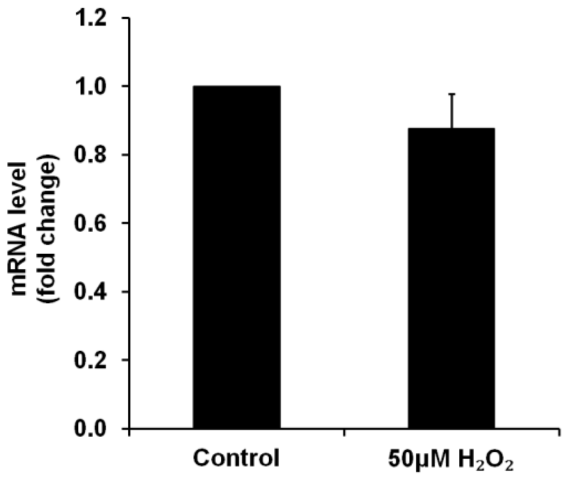

Supplementary Figure S2: Sub-lethal oxidative stress does not down-regulate TFEB expression. mRNA expression of TFEB was assessed in L6 myoblasts treated with 50µM H_2O_2 for 24h using SYBR Green Real-Time PCR, normalized to endogenous control 18s. Relative mRNA expression is expressed as fold change over untreated control cells. Comparative $\Delta\Delta C_t$ method was used to determine gene expression 24 hours following cells exposure to 50µM H_2O_2 . Values represent mean +/- SEM, n=3, $P > 0.05$ (t-test).

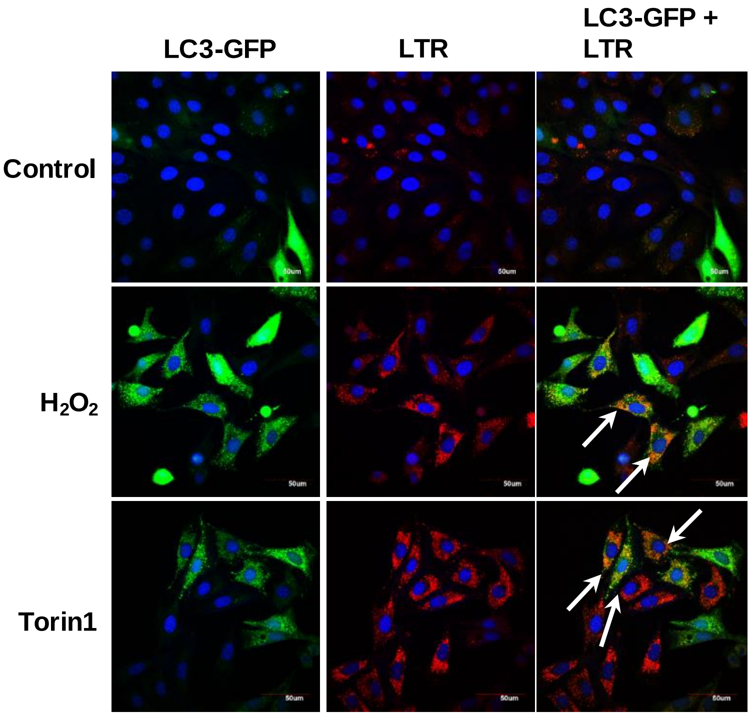

Supplementary Figure S3: Sub-lethal oxidative stress results in fusion of autophagosomes and lysosomes. Cells were transfected with LC3-GFP prior to treatment with 50μM H_2O_2 and 100nM Torin1 for 24h. Cells were then stained with LTR. Colocalization of LC3-GFP and LTR, indicating fusion of autophagosomes with lysosomes, was analyzed with confocal microscopy. Arrows on picture indicate overlapping of GFP-LC3 and LTR.

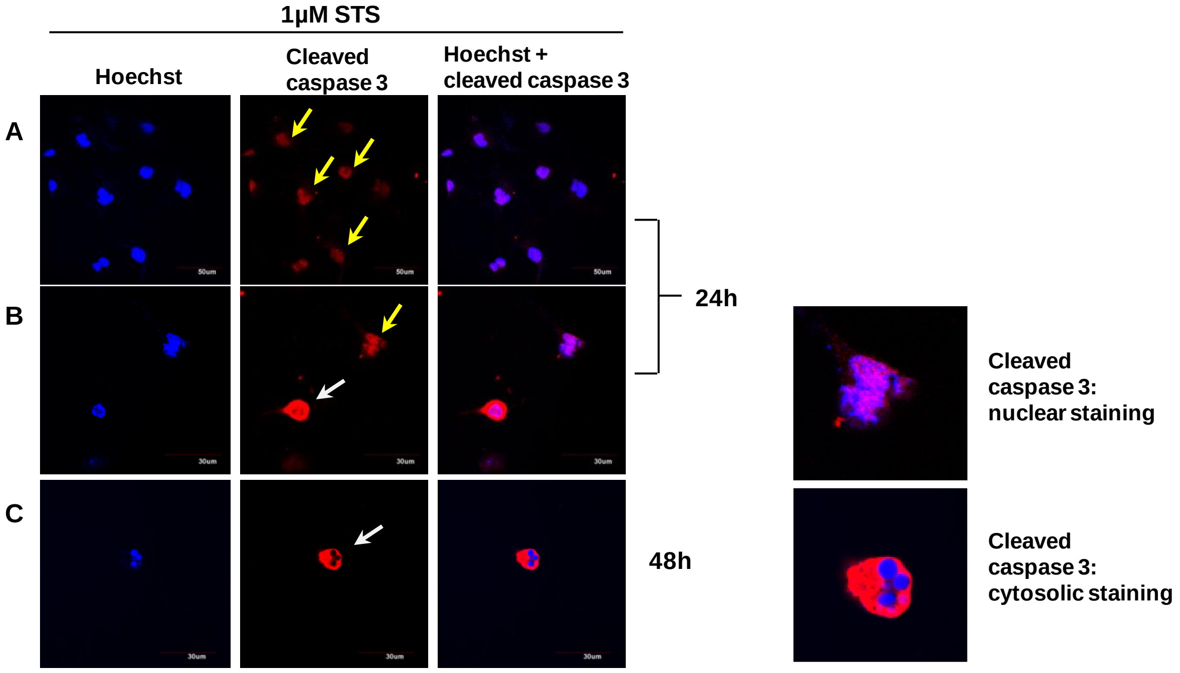

Supplementary Figure S4: **Cleaved caspase 3 shows cytosolic and nuclear localization in STS-treated cells**. Cleaved caspase 3 (red) immunofluorescence analysis of cells exposed to 1μM STS for (**A-B**) 24h and (**C**) 48h, as viewed under a confocal microscope. White arrows indicate cytosolic localization of cleaved caspase 3 while yellow arrow indicates a nuclear localization of cleaved caspase 3. (**A**) shows pictures in lower magnification (scale = 50μm), (**B-C**) in higher magnification (scale = 30μm).

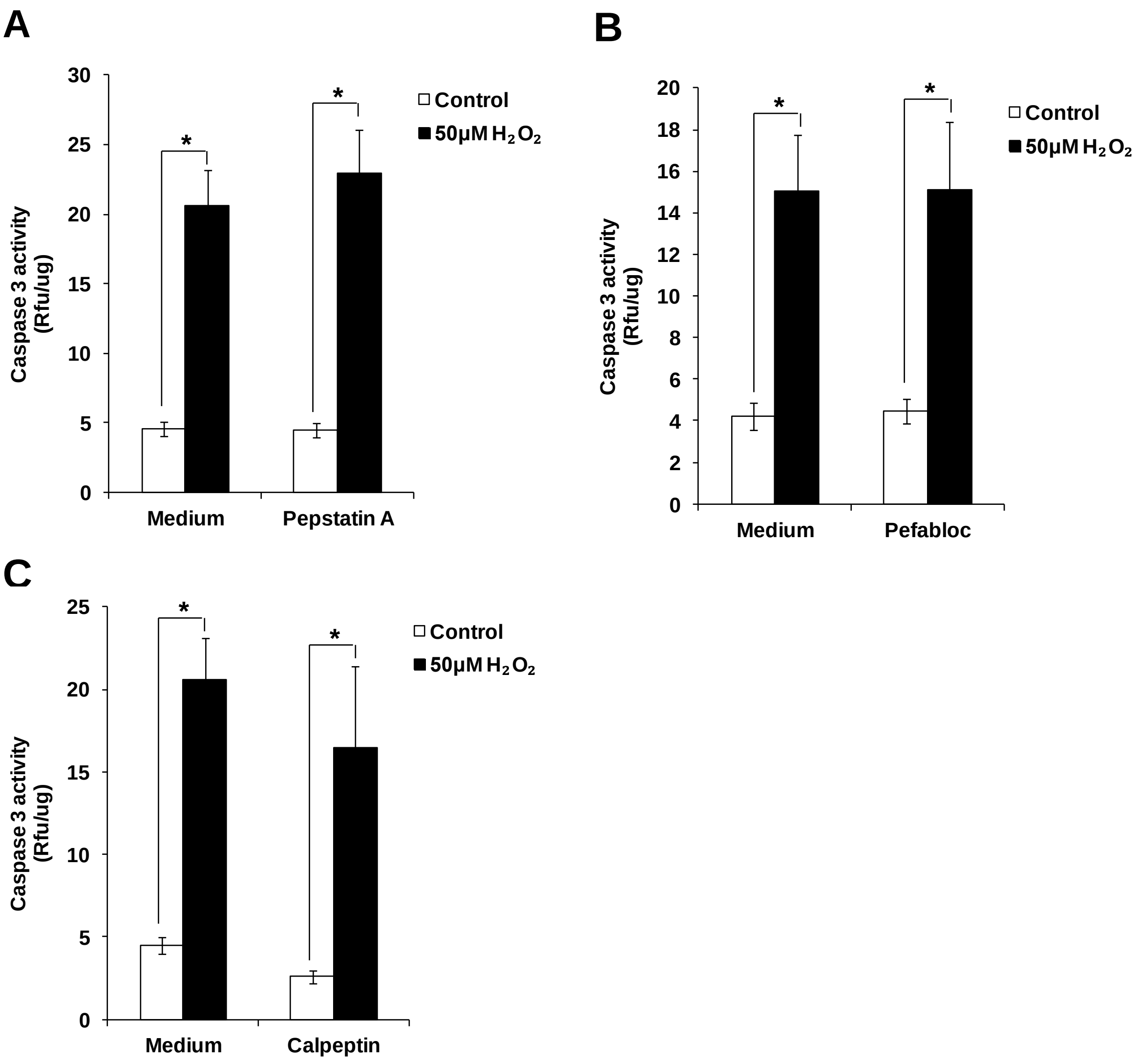

Supplementary Figure S5: Caspase 3 activation induced by sub-lethal oxidative stress is independent of serine proteases, aspartate proteases and calpain. Prior to treatment with 50μM H_2O_2, cells were pre-treated with (**a**) pepstatin A (100μM), (**b**) pefabloc (100μM), or (**c**) calpeptin (100μM). Cell lysate collected was assayed for caspase 3 activity with the fluorogenic substrate Ac-DEVD-AFC. Values represent mean + /- SEM, *$P < 0.05$, pepstatin A: n=3, pefabloc: n=4, calpeptin: n=3 (t-test). The effect of pepstatin A, pefabloc and calpeptine on the increase in caspase 3 activity was not statistically significant $P > 0.05$ (mixed model).

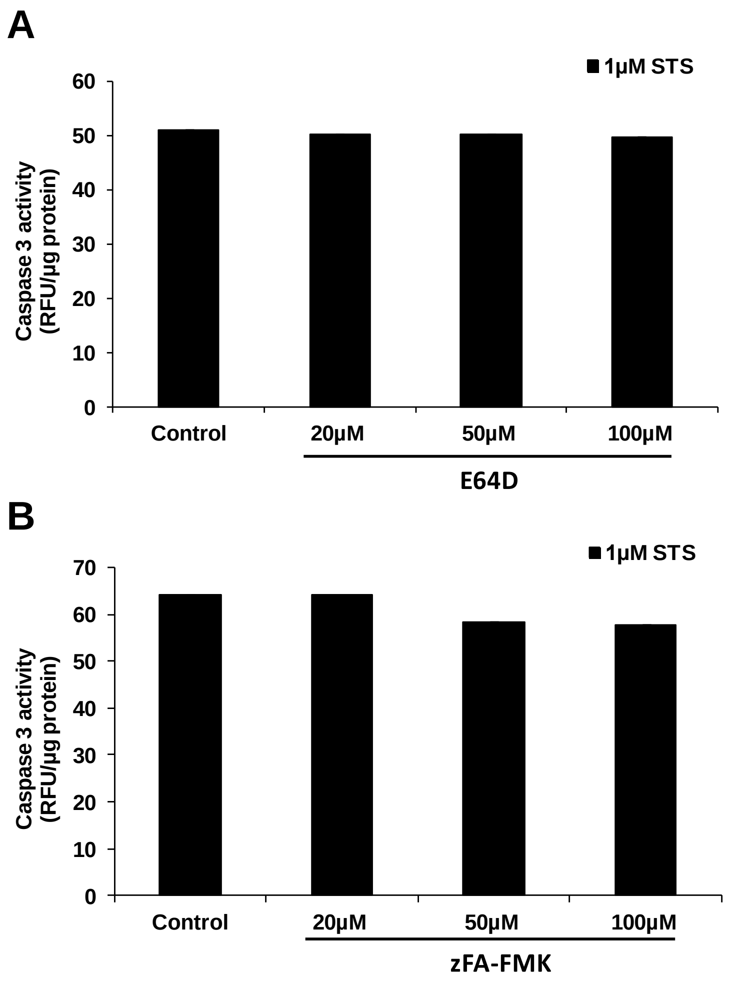

Supplementary Figure S6: Protease inhibitors E64D and zFA-FMK does not directly inhibit caspase 3 activity *in vitro*. STS-treated cell lysate with activated caspase 3 were loaded onto a 96-well plate and subsequently added with increasing dose of (**A**) E64D (**B**) zFA-FMK. Caspase 3 activity in cell lysate with or without the inhibitors was then assessed by fluorogenic substrate Ac-DEVD-AFC, n=1.

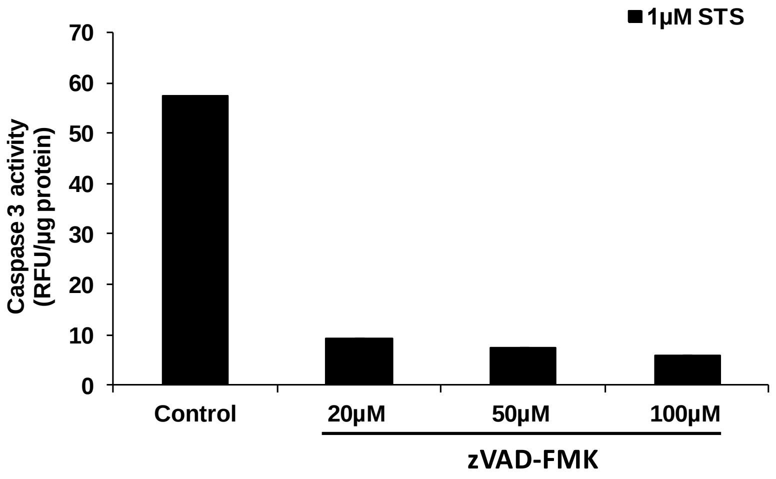

Supplementary Figure S7: Effect of zVAD-FMK on *in vitro* caspase 3 activitiy. STS-treated cell lysate with activated caspase 3 were loaded onto a 96-well plate and subsequently added with increasing dose of zVAD-FMK. Caspase 3 activity in cell lysate with or without the inhibitor was then assessed by fluorogenic substrate Ac-DEVD-AFC, n=1.

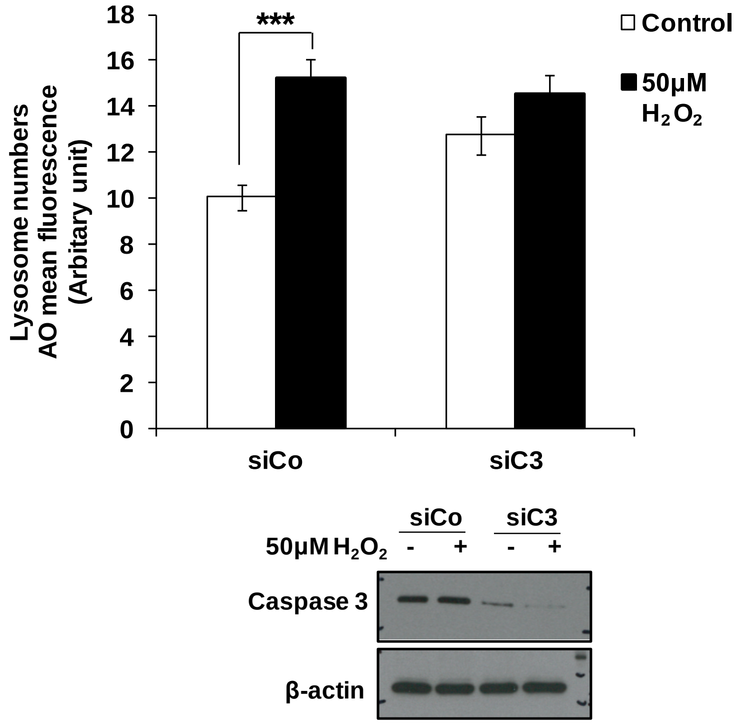

Supplementary Figure S8: Caspase 3 is involved in lysosome biogenesis upon sub-lethal oxidative stress. FACS analysis of AO staining in cells transfected with siC3 or negative control siRNA, 24h after 50μM H_2O_2 treatment. Values represent mean + /- SEM, ***P < 0.0005, n=6 (t-test).

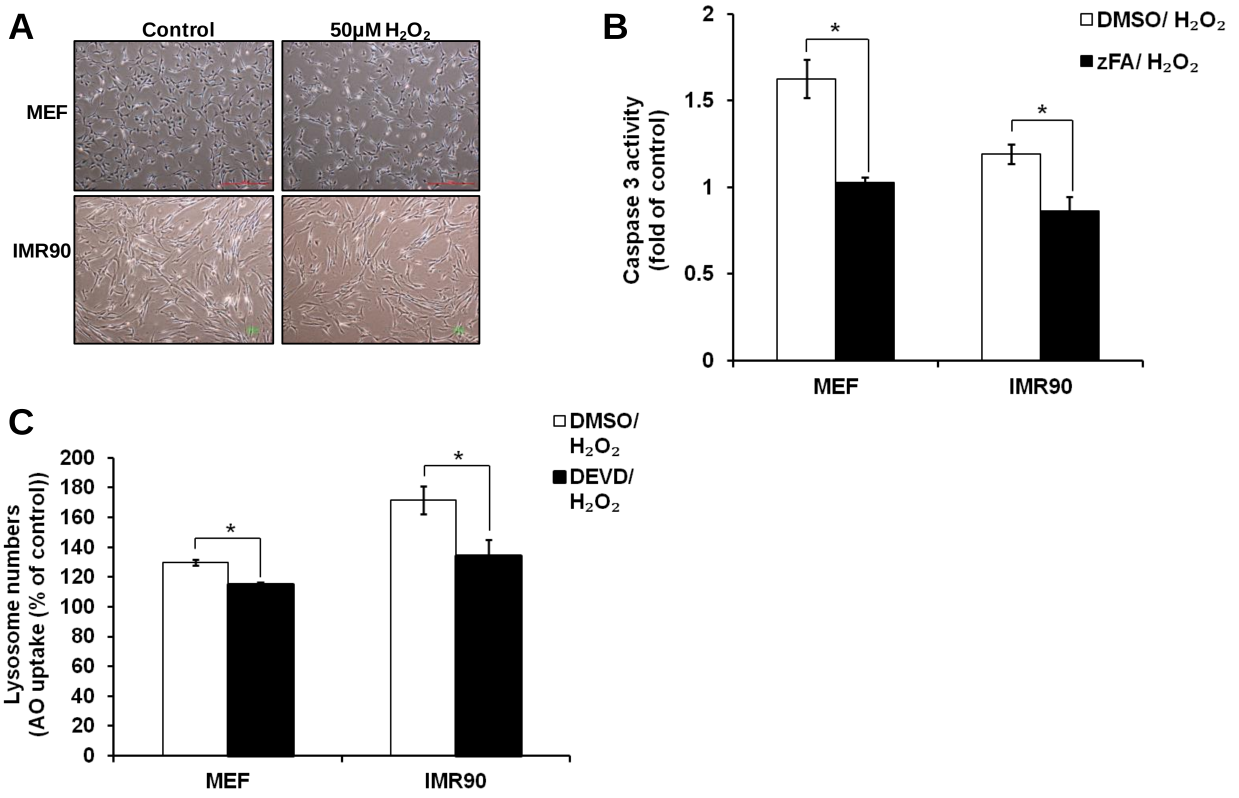

Supplementary Figure S9: Subtoxic oxidative stress activates the caspase 3-TFEB-lysosome biogenesis pathway in MEF and IMR90. (**A**) Cell morphology of MEF and IMR90 after 24h treatment of 50µM H_2O_2. (**B**) Caspase 3 activity in MEF and IMR90 cells pre-treated with 50µM zFA-FMK and exposed to 50µM H_2O_2 for 24h. Values represent mean +/- SEM, *$P < 0.05$; MEF: n=3, IMR90: n=4 (t-test). (**C**) Lysosome numbers in MEF and IMR90 pre-treated with 20µM zDEVD-FMK for 2h before being exposed to 50µM H_2O_2 for 24h, assessed by AO uptake assay. Values represent mean +/- SEM, *$P < 0.05$; n=4 (t-test).

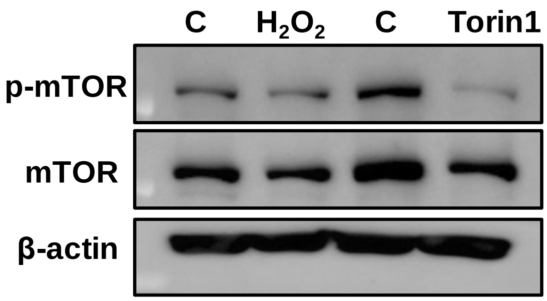

Supplementary Figure S10: Sub-lethal H_2O_2 treatment induces down-regulation of mTOR phosphorylation. L6 cells were treated with 50μM H_2O_2 and 100nM Torin1 24h. Cell lysate was harvested for analysis of mTOR phosphorylation (ser2448).

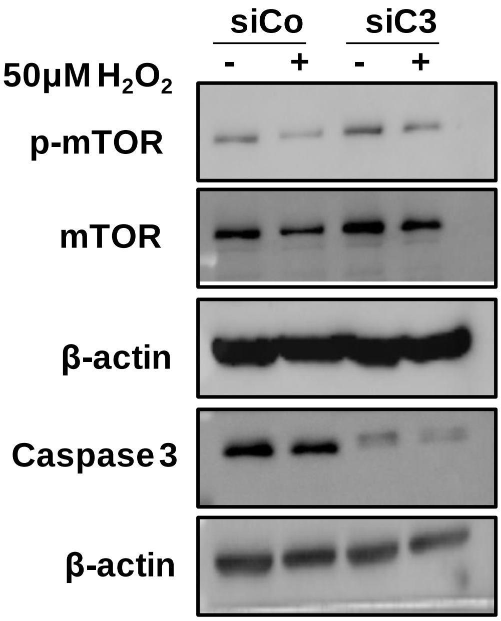

Supplementary Figure S11: Caspase 3 is not involved in down-regulation of mTOR phosphorylation. L6 cells were transfected with siC3 or siCo for 24h. After that, cells were treated with 50μM H_2O_2 for 24h. Cell lysate was harvested for analysis of mTOR phosphorylation (ser2448).

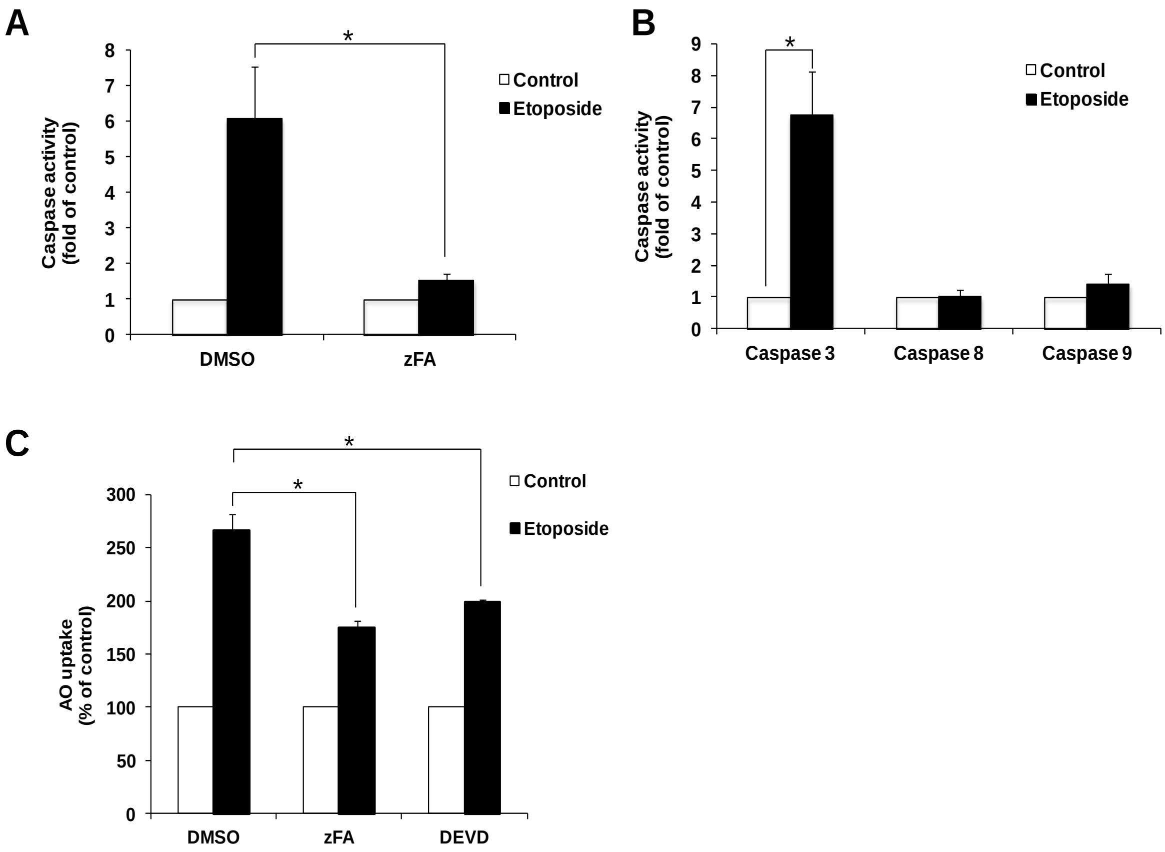

Supplementary Figure S12: Caspasae 3 regulation of lysosome biogenesis following cells exposure to etoposide. (A) L6 cells were pre-treated with 50µM zFA-FMK, before treatment with 150µM etoposide for 24h. Caspase 3 activity was assayed as described in Materials and Methods. (B) Caspase activity in L6 cells treated with 150µM etoposide for 24h. (C) FACS analysis of AO staining in L6 cells treated with 150µM etoposide in the presence of 50µM zFA-FMK or 20µM zDEVD-FMK.

Materials and Methods

Nuclear-cytoplasmic fractionation was performed with the NE-PER Nuclear and Cytoplasmic Extraction Reagents (PIERCE, Thermo Fisher Scientific Inc, Rockford, USA) according to manufacturer's protocol. Cell pellet was fully resuspended in Cytoplasmic Extraction Reagent I (CERI) by brief vortexing before Cytoplasmic Extraction Reagent II (CERII) was added. The cell suspension was vortexed followed by centrifugation and supernatant was collected as the cytosolic fraction. The nuclear pellet was then lysed in Nuclear Extraction Reagent (NER).

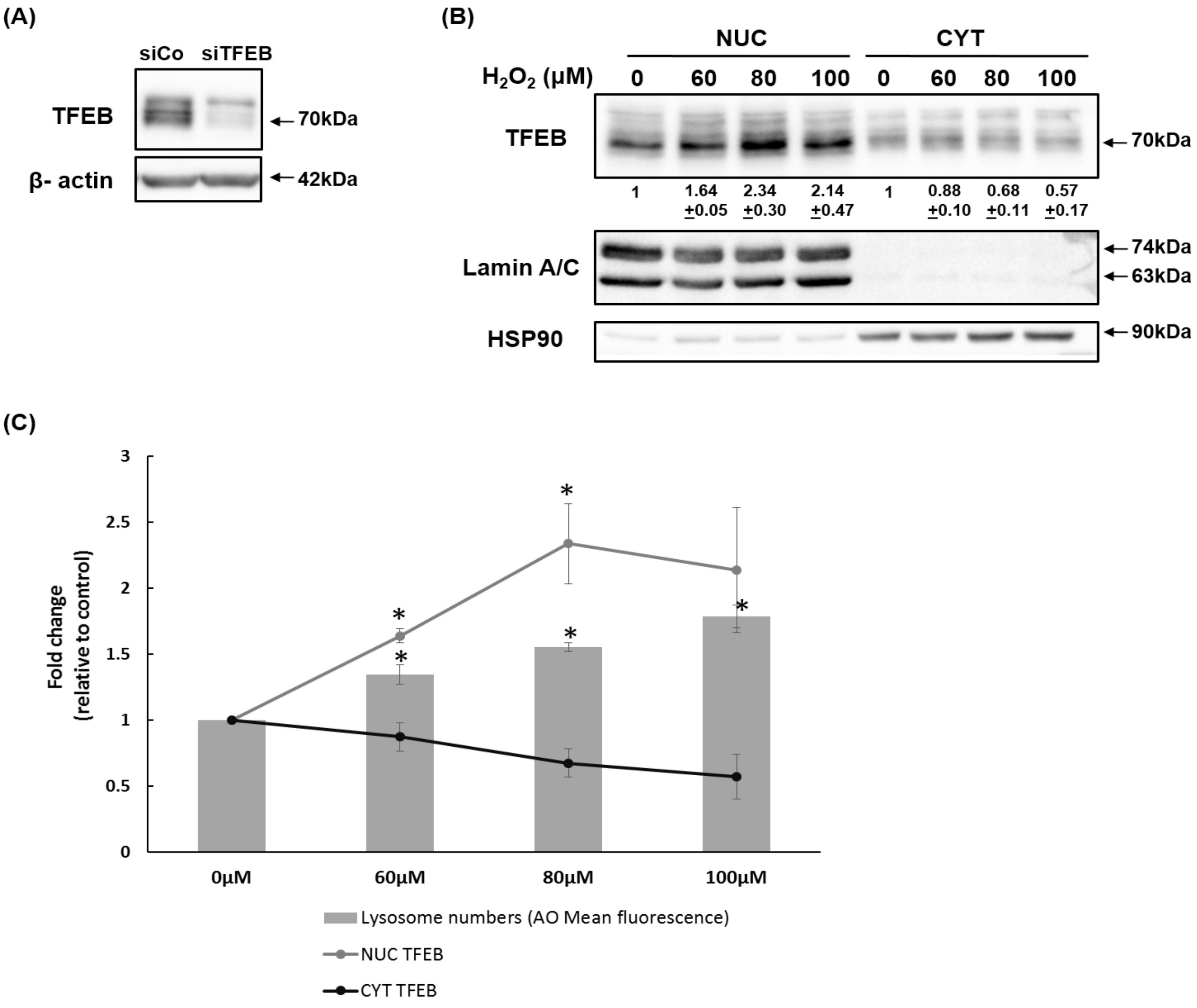

Supplementary Figure S13: (A) Validation of TFEB antibody (cell signaling #4240). L6 cells were transfected with siTFEB or negative control siRNA (siCo). Cells were harvested for Western Blot analysis 48h post transfection. **(B) Nuclear-cytoplasmic fractionation.** L6 cells were treated with increasing concentration of H_2O_2 for 24h and TFEB nuclear expression was assessed using Western Blot. NUC: nuclear fraction; CYT: cytoplasmic fraction. Band intensities of TFEB were quantified after normalization to loading control. The change in TFEB protein level in each fraction is shown as fold change relative to control cells. Values represent the mean +/- SE of three independent experiments. **(C)** Lysosomes number under the same condition as in

(B) was assessed using the AO uptake assay. Values represent mean of the fold change of AO uptake compared to control cells +/-SE of three independent experiments. Fold changes of TFEB in each fraction from (B) were also plotted on the same graph. * $P < 0.05$ as compared to control by t-test.

**Supplementary Table S1: Expression of selected TFEB gene targets following sublethal oxidative stress**		
Gene symbol (Mouse)	*Fold increase+/-SEM*	*Protein function*
**Lysosomal genes**		
Ctsb	1.90 +/- 0.21	Lysosomal cysteine protease
Lamp1	1.84 +/- 0.22	Lysosomal membrane glycoprotein
Mcoln1	1.84 +/- 0.36	Lysosomal cation channel
Ctsl	1.72 +/- 0.23	Lysosomal cysteine proteinase
Neu1	1.63 +/- 0.37	Lysosomal sialidase
Hexa	1.37 +/- 0.19	Alpha subunit of the lysosomal enzyme β-*hexosaminidase* A
Gba	1.17 +/- 0.26	Lysosomal house keeping enzyme
Lamp2	0.89 +/- 0.03	Lysosomal membrane glycoprotein
**Autophagy genes**		
Sqstm1	2.73 +/- 0.29	Ubiquitin and LC3 binding protein
Atg9b	1.64 +/- 0.18	Involved in autophagosome assembly and antisense transcript in the posttranscriptional regulation of eNOS3

Comparative $\Delta\Delta C_t$ method was used to determine gene expression 24 hours following cells exposure to 50µM H_2O_2. Expression levels were normalized to the expression levels of the housekeeping gene 18s. Fold change compared to untreated cells represent the average of at least 3 independent experiments. Results are shown as fold increase +/- SEM

**SupplementaryTable S2: Expression of selected TFEB gene targets following sublethal oxidative stress**		
Gene symbol (Human)	*Fold increase+/-SEM*	*Protein function*
**Lysosomal genes**		
CTSB	2.60 +/- 0.19	Lysosomal cysteine protease
NEU1	1.95 +/- 0.40	Lysosomal sialidase
LAMP1	1.89 +/- 0.25	Lysosomal membrane glycoprotein
GBA	1.88 +/- 0.14	Lysosomal house keeping enzyme
CTSL	1.60 +/- 0.15	Lysosomal cysteine proteinase
HEXA	1.60 +/- 0.22	Alpha subunit of the lysosomal enzyme β-*hexosaminidase* A
LAMP2	1.37 +/- 0.20	Lysosomal membrane glycoprotein
MCOLN1	1.17 +/- 0.08	Lysosomal cation channel
**Autophagy genes**		
SQSTM1	6.85 +/- 1.55	Ubiquitin and LC3 binding protein
ATG9B	1.54 +/- 0.13	Involved in autophagosome assembly and antisense transcript in the posttranscriptional regulation of eNOS3

Comparative $\Delta\Delta C_t$ method was used to determine gene expression 24 hours following cells exposure to 50µM H_2O_2. Expression levels were normalized to the expression levels of the housekeeping gene 18s. Fold change compared to untreated cells represent the average of at least 4 independent experiments. Results are shown as fold increase +/- SEM
